# 24-hour movement behaviours and cardiometabolic markers in women with polycystic ovary syndrome (PCOS): a compositional data analysis

**DOI:** 10.1093/humrep/deae232

**Published:** 2024-10-04

**Authors:** E Pesonen, V Farrahi, C J Brakenridge, M M Ollila, L C Morin-Papunen, M Nurkkala, T Jämsä, R Korpelainen, L J Moran, T T Piltonen, M Niemelä

**Affiliations:** Research Unit of Clinical Medicine, Department of Obstetrics and Gynecology, Oulu University Hospital, University of Oulu, Oulu, Finland; Research Unit of Health Sciences and Technology, University of Oulu, Oulu, Finland; Medical Research Center Oulu, Oulu University Hospital, University of Oulu, Oulu, Finland; Research Unit of Health Sciences and Technology, University of Oulu, Oulu, Finland; Institute for Sport and Sport Science, TU Dortmund University, Dortmund, Germany; Centre for Urban Transitions, Swinburne University of Technology, Melbourne, VIC, Australia; Active Life Lab, South-Eastern Finland University of Applied Sciences, Mikkeli, Finland; Research Unit of Clinical Medicine, Department of Obstetrics and Gynecology, Oulu University Hospital, University of Oulu, Oulu, Finland; Medical Research Center Oulu, Oulu University Hospital, University of Oulu, Oulu, Finland; Research Unit of Clinical Medicine, Department of Obstetrics and Gynecology, Oulu University Hospital, University of Oulu, Oulu, Finland; Medical Research Center Oulu, Oulu University Hospital, University of Oulu, Oulu, Finland; Medical Research Center Oulu, Oulu University Hospital, University of Oulu, Oulu, Finland; Department of Sports and Exercise Medicine, Oulu Deaconess Institute Foundation sr, Oulu, Finland; Research Unit of Population Health, University of Oulu, Oulu, Finland; Research Unit of Health Sciences and Technology, University of Oulu, Oulu, Finland; Medical Research Center Oulu, Oulu University Hospital, University of Oulu, Oulu, Finland; Medical Research Center Oulu, Oulu University Hospital, University of Oulu, Oulu, Finland; Department of Sports and Exercise Medicine, Oulu Deaconess Institute Foundation sr, Oulu, Finland; Research Unit of Population Health, University of Oulu, Oulu, Finland; Monash Centre for Health Research and Implementation, Monash University, Melbourne, VIC, Australia; Research Unit of Clinical Medicine, Department of Obstetrics and Gynecology, Oulu University Hospital, University of Oulu, Oulu, Finland; Medical Research Center Oulu, Oulu University Hospital, University of Oulu, Oulu, Finland; Research Unit of Health Sciences and Technology, University of Oulu, Oulu, Finland; Centre for Wireless Communications, University of Oulu, Oulu, Finland

**Keywords:** isotemporal substitution, metabolic diseases, PCOS, physical activity, sedentary behaviour, CoDA

## Abstract

**STUDY QUESTION:**

Are 24-h movement composition and time reallocations between the movement behaviours (moderate-to-vigorous physical activity (MVPA), light physical activity (LPA), sedentary behaviour (SB), and sleep) differentially associated with cardiometabolic markers in women with polycystic ovary syndrome (PCOS) relative to women without PCOS?

**SUMMARY ANSWER:**

There was no difference in 24-h movement composition between the groups, although among women without PCOS, reducing SB time while increasing either MVPA or LPA time was associated with beneficial differences in cardiometabolic markers, whereas in women with PCOS beneficial differences were observed only when SB time was replaced with MVPA.

**WHAT IS KNOWN ALREADY:**

Women with PCOS display lower levels of physical activity, higher sedentary time, and less total sleep than women without the syndrome. Exercise interventions among women with PCOS have shown improvements in body composition and insulin sensitivity, while the findings regarding blood pressure, insulin resistance, and lipid profiles are contradictory.

**STUDY DESIGN, SIZE, DURATION:**

This study was part of a prospective, general population-based Northern Finland Birth Cohort 1966 (NFBC1966) (n = 5889 women). At the 31-year and 46-year follow-up, data collection was performed through postal and clinical examinations, including fasting blood samples and anthropometric measurements. Accelerometer data collection of 14 days (n = 2602 women) and a 2-h oral glucose tolerance test (n = 2780 women) were performed at the 46-year follow-up. Participants were identified as women with or without PCOS at age 31 (n = 1883), and the final study population included those who provided valid accelerometer data at age 46 (n = 857).

**PARTICIPANTS/MATERIALS, SETTING, METHODS:**

Women with PCOS (n = 192) were identified based on the 2023 International Evidence-based Guideline, while those who exhibited no PCOS features were considered women without PCOS (controls; n = 665). Accelerometer-measured MVPA, LPA, and SB were combined with self-reported sleep to obtain 24-h compositions. Multivariable regression analysis based on compositional data analysis and isotemporal reallocations were performed to investigate the associations between 24-h movement composition and cardiometabolic markers. Isotemporal reallocations were expressed as differences (%Δ) from the sample’s mean.

**MAIN RESULTS AND THE ROLE OF CHANCE:**

There was no difference in overall 24-h movement composition between women with PCOS and controls in midlife. The 24-h movement composition was associated with waist circumference, triglycerides, fasting serum insulin, and Homeostatic Model Assessment–insulin resistance (HOMA-IR) in both controls and women with PCOS. Reallocating 15 min from SB to MVPA was associated with favourable differences in cardiometabolic markers in both controls (%Δ range from −1.7 to −4.9) and women with PCOS (%Δ range from −1.9 to −8.6). Reallocating 15 min from SB to LPA was also associated with favourable differences in cardiometabolic markers among controls (%Δ range from −0.5 to −1.6) but not among women with PCOS.

**LIMITATIONS, REASONS FOR CAUTION:**

The substitution technique used in this study is theoretical, which can be considered as a limitation. Other limitations of this study are the use of self-reported sleeping time and the difference in the group sample sizes.

**WIDER IMPLICATIONS OF THE FINDINGS:**

These findings suggest that women with PCOS should be targeted with interventions involving physical activity of at least moderate intensity to improve their cardiometabolic health and underline the importance of developing tailored activity guidelines for women with PCOS.

**STUDY FUNDING/COMPETING INTEREST(S):**

This study was funded by the Jenny and Antti Wihuri Foundation, Sigrid Juselius Foundation, Novo Nordisk (NNF21OC0070372), Research Council of Finland (315921/2018, 321763/2019, 6GESS 336449), Ministry of Education and Culture of Finland (OKM/54/626/2019, OKM/85/626/2019, OKM/1096/626/2020, OKM/20/626/2022, OKM/76/626/2022, and OKM/68/626/2023), and Roche Diagnostics International Ltd. L.J.M. is supported by a Veski Fellowship. M.Nu. has received funding from Fibrobesity-project, a strategic profiling project at the University of Oulu, which is supported by Research Council of Finland (Profi6 336449). NFBC1966 follow-ups received financial support from University of Oulu (Grant no. 65354, 24000692), Oulu University Hospital (Grant no. 2/97, 8/97, 24301140), Ministry of Health and Social Affairs (Grant no. 23/251/97, 160/97, 190/97), National Institute for Health and Welfare, Helsinki (Grant no. 54121), Regional Institute of Occupational Health, Oulu, Finland (Grant no. 50621, 54231), and ERDF European Regional Development Fund (Grant no. 539/2010 A31592). T.T.P. declares consulting fees from Gedeon Richter, Organon, Astellas, Roche; speaker’s fees from Gedeon Richter, Exeltis, Roche, Stragen, Merck, Organon; and travel support from Gedeon Richter. The remaining authors declare no conflicts of interest.

**TRIAL REGISTRATION NUMBER:**

N/A.

## Introduction

Polycystic ovary syndrome (PCOS) is the most common endocrinopathy, affecting up to 18% of reproductive-aged women (March *et al.*, 2010; Bozdag *et al.*, 2016). For a PCOS diagnosis, at least two of the following criteria must be met: (i) irregular menstrual cycle or ovulatory dysfunction, (ii) clinical or biochemical hyperandrogenism (HA), and (iii) polycystic ovarian morphology (PCOM) (Teede *et al.*, 2023). According to the 2023 International Evidence-based Guideline for the assessment and management of PCOS, excess anti-Müllerian hormone (AMH) levels can be used as a surrogate for PCOM (Teede *et al.*, 2023). Even though women with PCOS seem to have a good prognosis on achieving at least one child (Joham *et al.*, 2014; West *et al.*, 2014; Persson *et al.*, 2019), the syndrome is associated with adverse reproductive outcomes, such as reduced fecundity (Persson *et al.*, 2019) and an increased risk of pregnancy complications, such as miscarriage, gestational diabetes, and pre-eclampsia (Bahri Khomami *et al.*, 2019). Additionally, women with PCOS are prone to a wide range of adverse health outcomes (Kujanpää *et al.*, 2024), including metabolic, psychological, and sleep-related disturbances (Stener-Victorin *et al.*, 2024). Although weight loss attempts are common in this population (Pesonen *et al.*, 2023), up to 70% of women with PCOS have pre-obesity or obesity, which further exacerbates unfavourable cardiometabolic marker levels (Ollila *et al.*, 2016, 2017) and contributes to a chronic inflammatory state (Rudnicka *et al.*, 2021). Insulin resistance and compensatory hyperinsulinemia are key etiologic features of PCOS, which are independent of, but aggravated by, obesity (Baptiste *et al.*, 2010; Diamanti-Kandarakis and Dunaif, 2012). Indeed, 75–95% of women with PCOS exhibit insulin resistance (Stepto *et al.*, 2013), and BMI-matched studies have shown that their risk of metabolic syndrome is two times higher than that of women without PCOS (Moran *et al.*, 2010; Lim *et al.*, 2019).

In the general population, physical activity is a well-established method for the prevention and management of cardiometabolic diseases (Kraus *et al.*, 2019). In women with PCOS, improvements through exercise interventions have been observed in terms of cardiorespiratory fitness (Kite *et al.*, 2019; Patten *et al.*, 2020; Breyley-Smith *et al.*, 2022), body composition (Benham *et al.*, 2018; Kite *et al.*, 2019; Kelly dos Santos *et al.*, 2020; Patten *et al.*, 2020; Breyley-Smith *et al.*, 2022), and insulin sensitivity (Benham *et al.*, 2018; Kite *et al.*, 2019; Patten *et al.*, 2020), while the findings regarding blood pressure, glucose levels, insulin resistance, and lipid profile are contradictory (Benham *et al.*, 2018; Kite *et al.*, 2019; Patten *et al.*, 2020; Breyley-Smith *et al.*, 2022). Importantly, physical activity provides health benefits also in the absence of weight loss (Hutchison *et al.*, 2011; Sabag *et al.*, 2022) and potentially helps to restore ovulation in women with PCOS (Palomba *et al.*, 2008; Mena *et al.*, 2019). However, due to the limited number of good-quality randomized controlled trials, the relatively small number of participants across studies, and the considerable variation in exercise protocols between studies, the existing synthesized evidence on the relationship between physical activity and PCOS is scarce. Consequently, it remains uncertain whether one form of exercise is superior to another in the management of PCOS (Colombo *et al.*, 2023). The recommendations of international evidence-based guidelines for PCOS are currently the same as those for the general population: 150–300 min of moderate-intensity exercise or 75–150 min of vigorous-intensity exercise per week (Teede *et al.*, 2023). Thus, tailored physical activity guidelines for the management of PCOS have yet to be developed (Stepto *et al.*, 2019).

The current available evidence, primarily based on self-reported data, suggests women with PCOS exhibit lower total levels of physical activity and higher sedentary time than women without PCOS (Tay *et al.*, 2020; Kazemi *et al.*, 2022). Moreover, they have more sleep disturbances and less objectively measured total sleep than women without the syndrome (Oberg *et al.*, 2023). Indeed, unfavourable lifestyle behaviours potentially increase the likelihood of cardiometabolic health deterioration in women with PCOS compared to the general population (Ekelund *et al.*, 2019a; Krittanawong *et al.*, 2019). However, critical knowledge gaps remain. Previous studies on PCOS have examined physical activity, sedentary behaviour (SB), and sleep separately, ignoring the codependent nature of these behaviours and potentially leading to biased findings (Chastin *et al.*, 2015). Furthermore, there is a lack of device-based activity measurements among women with PCOS, although they are needed to comprehensively assess the entire spectrum of movement behaviours across the whole day (Prince *et al.*, 2008; Chastin *et al.*, 2015). Indeed, emerging evidence has clearly shown the importance of considering movement behaviours within the entire 24-h period when investigating their associations with health outcomes (Chastin *et al.*, 2015; Dumuid *et al.*, 2018; McGregor *et al.*, 2019).

Movement behaviours, i.e. moderate-to-vigorous physical activity (MVPA), light physical activity (LPA), SB, and sleep, are mutually exclusive parts of the 24-h day (Dumuid *et al.*, 2018). Appropriate statistical methods are required to identify the interrelationships between these behaviours. Compositional data analysis (CoDA), a well-established method in various fields, has also been demonstrated to be suitable for health behaviour research (Chastin *et al.*, 2015; Dumuid *et al.*, 2018). CoDA considers daily movement behaviours to be interrelated and reflects the results as time exchanges: time spent on one behaviour decreases the time spent on other behaviours (Chastin *et al.*, 2015). Previous studies using CoDA have found that replacing SB time with MVPA or LPA is associated with better cardiometabolic health (Farrahi *et al.*, 2021) and that decreasing SB while increasing MVPA reduces the likelihood of all-cause mortality in the general population (Niemelä *et al.*, 2023). However, there is no prior information on how movement behaviours should be reallocated across the 24-h cycle to be beneficially associated with cardiometabolic markers in women with PCOS.

To fill the knowledge gap, we examined accelerometer-measured physical activity and SB as well as self-reported sleep in a 24-h cycle in a population-based cohort of women with and without PCOS. Our aim was to determine whether the 24-h movement composition and time reallocations between these behaviours are differentially associated with cardiometabolic markers in women with PCOS relative to women without PCOS in midlife.

## Materials and methods

### Study population

The data used in this study were derived from the Northern Finland Birth Cohort 1966 (NFBC1966), a general population-based birth cohort. The NFBC1966 includes all individuals who were expected to be born in the two northernmost provinces of Finland in 1966 (N = 12 231; women n = 5889). Since they were born, the participants have been followed regularly across their life courses. Detailed cohort descriptions and follow-up protocols have been published previously (Nordström *et al.*, 2022; University of Oulu). This study included participants who were identified as women with or without PCOS at the 31-year follow-up (during years 1996–1997) and who provided valid accelerometer data at the 46-year follow-up (during years 2012–2014). Data collection at age 46 was performed through postal questionnaires regarding health and lifestyle, clinical examinations (fasting blood samples and anthropometric measurements), and 75-g oral glucose tolerance tests (OGTTs).

At age 31, women with PCOS were identified according to the 2023 International Evidence-based Guideline for the assessment and management of PCOS (Teede *et al.*, 2023) as those who met at least two of the following criteria: (i) irregular or absent menstrual cycle (oligomenorrhoea or amenorrhoea [OA]), (ii) clinical or biochemical HA, or (iii) AMH levels exceeding 3.2 ng/ml (surrogate for polycystic ovary morphology). A flowchart of the participant inclusion process is presented in Supplementary Fig. S1. Briefly, women who reported having been diagnosed with PCOS in the 46-year follow-up but who did not meet the PCOS criteria at age 31 (n = 19) were excluded from the analysis. Pregnant women and women using hormonal contraceptives (n = 1020) at age 31 and those who did not consent to the use of their data were excluded. Women who did not fulfil any PCOS criteria at age 31 were classified as women without PCOS (controls). The formation of the study population has been reported in more detail elsewhere (Piltonen *et al.*, 2023). Women with PCOS were also categorized into the following phenotypes: A-phenotype (HA + OA + AMH, n = 40), B-phenotype (HA + OA, n = 22), C-phenotype (HA + AMH, n = 62), and D-phenotype (OA + AMH, n = 68). We combined A- and B-phenotypes as ‘the classic A + B-phenotype’ (HA + OA with or without elevated AMH, n = 62) (Mumusoglu and Yildiz, 2020). Women with PCOS were also compared with each other based on the presence or absence of hyperandrogenism: HA-phenotype (n = 124) and non-HA-phenotype (n = 68).

### Ethical approval

This study was approved by the Ethics Committee of the Northern Ostrobothnia Hospital District in Oulu, Finland (94/2011). All participants provided informed consent. The participants’ anonymity was ensured by encrypting identity information and using identification codes.

### Measurements

#### 24-h movement behaviours

The participants were asked to use a waist-worn accelerometer (Hookie AM20; Traxmeet Ltd., Espoo, Finland) for 14 consecutive days during all waking hours, except during water-based activities. Raw acceleration data were collected at 100 Hz and segmented into 6-s epochs. The mean amplitude deviation (MAD) was calculated for each 6-s window (Farrahi *et al.*, 2021), and monitor non-wear time intervals were identified and removed using a method closely resembling a validated and widely accepted method for processing count-based accelerometer data (Choi *et al.*, 2011). Monitor wear time epochs that were found to overlap with self-reported sleep times were discarded. Monitor-wearing times were classified into SB (<1.5 metabolic equivalents of a task [MET] in sitting or lying position), LPA (1.5–3.0 MET; light activity and standing still), or MVPA (≥3 MET; e.g. brisk walking) based on the MAD value cut-points and a validated algorithm for posture detection in hip accelerometer data (Vähä-Ypyä *et al.*, 2015, 2018). The time spent on each category (in minutes per day) was calculated, and the averages were computed over all valid days. A valid measurement day was defined as at least 10 h of device-wearing time. A minimum of four valid days was required for a participant to be included in the study. The accelerometer data collection and analysis have been reported in more detail previously (Farrahi *et al.*, 2021). Sleep duration was self-reported in response to the question, ‘How many hours do you sleep, on average, per day?’ The responses were converted to minutes of sleep per day.

#### Questionnaires and clinical measurements

Questionnaires were used to obtain information on lifestyle, health, and sociodemographic factors. These included smoking status, alcohol consumption, education, marital status, medication use (blood glucose lowering, lipid modifying, or antihypertensive), and psychological distress (depression and anxiety) assessed using the Hopkins Symptoms Checklist-25. A Healthy Diet Index, based on an existing validated index (Lindström *et al.*, 2021), was derived from the food frequency questionnaire with 38 questions and 6 response categories (ranging from ‘less than once a month or not at all’ to ‘once a day or more’), and the questions on meal rhythm, dairy products, types of fat used, and bread consumption. It includes seven subdomains: meal pattern (10%), grains (20%), fruits and vegetables (20%), fats (15%), fish and meat (10%), dairy (10%), and snacks and treats (15%). Scores range from 0 to 100, with higher scores indicating better dietary quality. The content of the Healthy Diet Index with more details has been described in Supplementary Table S1.

During the clinical examinations, weight (in kilograms) was measured using a regularly calibrated digital scale, and height (in centimetres) was measured twice with a standard calibrated stadiometre. Based on these measurements, the BMI was calculated as weight in kilograms divided by the square of height in metres. Waist circumference was measured by trained nurses from the midway point between the iliac crest and the lowest ribs. Systolic blood pressure (SBP) and diastolic blood pressure (DBP) were measured three times in a seated position after 15 min of rest, and the measurements were averaged (Omron Digital Automatic Blood Pressure Monitor Model M10-IT; Omron, Kyoto, Japan). The mean arterial pressure (MAP) was calculated as DBP + 1/3 (SBP – DBP).

#### Laboratory methods

After overnight fasting, venous blood samples were drawn, and the samples were analysed in NordLab Oulu, a testing laboratory (T113) accredited by the Finnish Accreditation Service (EN ISO 15189). The serum triglycerides levels were determined by using enzymatic assay methods, and fasting plasma glucose (fp-glucose) was analysed by an enzymatic dehydrogenase method (Advia 1800; Siemens Healthcare Diagnostics Inc., Tarrytown, NY, USA). Two fp-glucose values exceeding 11 mmol/l among women with PCOS were excluded. Fasting serum insulin (fs-insulin) was analysed by a chemiluminometric immunoassay (Advia Centaur XP, Siemens Healthcare Diagnostics, Tarrytown, New York, USA). The Homeostatic Model Assessment–insulin resistance (HOMA-IR) index was calculated with fp-glucose and fs-insulin values (fp-glucose × fs-insulin/22.5). High-sensitivity C-reactive protein (hs-CRP) was analysed by an immune-nephelometric assay (BN ProSpec, Siemens Healthcare Diagnostics, Newark, Delaware, USA); values over 10 (mg/l) were excluded from this study. Additionally, there was an OGTT from which 2-h plasma glucose and insulin levels were obtained.

Testosterone, sex hormone-binding globulin (SHBG) and AMH values from 31-year follow-up measurements were used to identify women with PCOS, as previously described (Ollila *et al.*, 2016; Piltonen *et al.*, 2023). Briefly, testosterone was analysed using Agilent triple quadrupole 6410 liquid chromatography-mass spectrometry equipment (Agilent Technologies, Inc., Wilmington, DE, USA). SHBG was assayed by fluoroimmunoassay (Wallac, Inc. Ltd, Turku, Finland). The free androgen index was calculated as follows: 100 × testosterone (nmol/l)/SHBG (nmol/l). Serum AMH was measured using the automated Elecsys electrochemiluminescence immunoassay on a cobas e 411 analyser (Roche Diagnostics, Germany).

### Statistical analysis

The statistical analyses were performed using R version 4.1.2 (R Core Team, Vienna, Austria) and IBM SPSS Statistics version 28.0.1 (IBM Corp., Armonk, NY, USA). Descriptive data were reported as medians with 25th and 75th percentiles and prevalences (%). Values of *P* < 0.05 were considered statistically significant. CoDA was performed using the ‘compositions’ and ‘deltacomp’ packages in R according to prior research (Dumuid *et al.*, 2018; Brakenridge *et al.*, 2021). For each participant, the compositional mean was determined by rescaling the geometric means of the behaviours to sum them up to 1440 min (24 h). As time-use data cannot be directly included in linear regression models due to perfect collinearity (Hron *et al.*, 2012), each participant’s MVPA, LPA, SB, and sleep times were transformed into isometric log-ratio (ilr) coordinates, which represent the importance of one behaviour (e.g. MVPA) relative to the geometric mean of the other behaviours (e.g. SB, LPA, and sleep). To examine intergroup differences in movement behaviours, the geometric means were computed as a log-ratio (women with PCOS/controls) and then bootstrapped to calculate the percentage difference with 95% CIs as described previously (Gupta *et al.*, 2018; Brakenridge *et al.*, 2021). The intergroup difference in the overall 24-h movement composition was determined using Hotelling’s *T*^2^ test (‘Hotelling’ package in R).

Multiple linear regression was used to investigate the associations between 24-h movement behaviours and cardiometabolic markers. All dependent variables were log-transformed to improve the normality of the residuals. MVPA, LPA, SB, and sleep times were entered into separate models for each cardiometabolic marker. The ilr coordinates were entered as independent variables, and the associations were tested in unadjusted and adjusted models. In Model 1, smoking, alcohol consumption, marital status, education, medication use, and psychological distress were used as possible covariates based on previous studies (Ollila *et al.*, 2017; Tay *et al.*, 2020), while Model 2 was further adjusted with the Healthy Diet Index to separately examine the role of dietary quality. Furthermore, as a secondary analysis, we conducted a Model 3 with additional adjustment with BMI to examine whether differences in cardiometabolic outcomes were mediated by variation in weight. In the regression models, the entire 24-h composition served as an exposure variable, and the coefficient β of the ilr1 coordinate reflected the effect of the time spent on one behaviour relative to the other three (Farrahi *et al.*, 2021). The requirements for model residuals were examined, although linear associations between MVPA, LPA, SB, and cardiometabolic markers were assumed. Furthermore, an interaction analysis was performed by including an interaction variable between the ilr-transformed variables (MVPA, LPA, SB, and sleep) and the group variable (0 = controls, 1 = women with PCOS) in the models. We also conducted a sub-group analysis, in which we examined the univariate associations between 24-h movement composition and cardiometabolic markers in PCOS-phenotypes.

We tested the possibility of a U-shaped relationship regarding sleep; if the association between sleep duration and cardiometabolic marker in regression models was significant (*P* < 0.05), the association was considered linear. The models that had an insignificant association were rerun after including a quadratic term for sleep duration. When the quadratic term was significant (*P* < 0.10), the relationship between sleep duration and the cardiometabolic marker was considered U-shaped, and the analysis for the cardiometabolic marker was stratified by mean sleep duration (<8.5 and ≥8.5 h/day) (Simonsohn, 2019).

The second part of the analyses consisted of a pairwise isotemporal substitution model aimed at exploring the theoretical differences in cardiometabolic marker estimates when time was reallocated from one movement behaviour to another. The theoretical differences in cardiometabolic outcomes resulting from time reallocations were estimated using adjusted model including smoking, alcohol consumption, marital status, education, medication use, and psychological distress as covariates. Isotemporal substitution was performed only for cardiometabolic markers that showed significant associations with the overall 24-h movement composition in univariate analyses among both groups. The reallocated time was added to or subtracted from the mean behaviour composition in intervals to determine the dose–response associations between time spent on movement behaviours and cardiometabolic markers (Chastin *et al.*, 2021). The time reallocations from and to MVPA ranged from 5 to 15 min to maintain below the mean values of MVPA and ensure realistic time exchanges. The time reallocations between LPA, SB, and sleep ranged from 15 to 45 min due to their higher mean values. Time reallocations of 15 min represent ∼1% time use in a 24-h cycle and are in line with the approaches used in previous CoDA studies, enabling a meaningful interpretation of the results (Chastin *et al.*, 2021; Niemelä *et al.*, 2023). The estimated differences were back-transformed to express the results as percentage differences from the original mean values, following the methodology used in prior studies (Brakenridge *et al.*, 2021; Farrahi *et al.*, 2021). CIs were determined according to the standard error of the delta estimate.

## Results

### Characteristics of the study population

A total of 665 controls and 192 women with PCOS provided valid accelerometer data in addition to self-reported sleep durations. The characteristics of the study population are presented in Table 1. As expected, women with PCOS had more adverse cardiometabolic profiles than women without PCOS, as indicated by their significantly higher waist circumference, triglyceride, fs-insulin, HOMA-IR, 2-h glucose, 2-h insulin, and MAP values. Women with PCOS also reported higher rates of medication use (blood glucose lowering, lipid modifying, or antihypertensive).

**Table 1.** Characteristics of the study population (n* *=* *857).

	N	Control (n* *=* *665)	N	PCOS (n* *=* *192)	*P*-value
**Anthropometry**					
Weight (kg)	665	68.0 [61.4; 79.9]	192	71.7 [63.3; 84.2]	**0.009**
BMI (kg/m^2^)	665	25.1 [22.6; 28.9]	192	26.6 [23.8; 30.9]	**0.001**
**Sociodemographic**					
Education	664		191		0.233
Basic		27 (4)		6 (3)	
Secondary		410 (62)		131 (69)	
Tertiary		227 (34)		54 (28)	
Married/co-habiting	661	515 (78)	191	153 (80)	0.517
**Behavioural**					
Alcohol use (g/d)	665	2.9 [0.6; 8.2]	192	2.3 [0.4; 7.6]	0.052
Smoking	665		192		0.864
No smoker		396 (60)		117 (61)	
Former smoker		150 (23)		45 (23)	
Current smoker		114 (17)		30 (16)	
Dietary quality scores	649	54.5 ± 9.99	190	54.6 ± 10.1	0.921
**Psychological distress**	625	1.28 [1.12; 1.48]	180	1.28 [1.12; 1.48]	0.796
**Blood glucose, lipid, or hypertension medication**	665	93 (14)	192	39 (20)	**0.032**
Blood glucose lowering	582	7 (1)	171	6 (4)	
Lipid modifying	665	18 (3)	192	5 (3)	
Antihypertensive	665	81 (12)	192	36 (19)	
**Cardiometabolic markers**					
Waist circumference (cm)	663	83.5 [77.0; 94.0]	191	88.0 [80.5; 99.0]	**<0.001**
Triglycerides (mmol/l)	665	1.01 [0.71; 1.26]	192	1.02 [0.73; 1.39]	**0.029**
fp-glucose (mmol/l)	656	5.3 [5.0; 5.6]	186	5.3 [5.0; 5.7]	0.314
fs-insulin (mU/l)	660	7.1 [4.9; 10.4]	187	7.8 [5.6; 11.8]	**0.014**
HOMA-IR	634	0.96 [0.67; 1.38]	180	1.06 [0.75; 1.57]	**0.013**
2-h glucose (mmol/l)	586	5.5 [4.7; 6.4]	163	5.7 [4.8; 7.0]	**0.020**
2-h insulin (mU/l)	584	41.6 [28.4; 61.7]	165	45.2 [33.1; 70.9]	**0.023**
hs-CRP (mg/l)	654	0.70 [0.36; 1.55]	186	0.80 [0.37; 1.84]	0.517
MAP (mmHg)	661	94 [87; 102]	191	96 [89; 104]	**0.011**

Continuous data are reported as median and 25th and 75th percentiles, and categorical data as number of cases (percentage of the stratified population). Statistically significant differences between controls and women with PCOS (*P *<* *0.05) are bolded. Dietary quality was assessed with a Healthy Diet Index (scores 0–100). Psychological distress was assessed by using the Hopkins Symptoms Checklist-25 (HSCL-25), the total score cut-off point ≥1.75 indicates a psychiatric disorder (depression/anxiety). Blood glucose-lowering drugs included ATC-codes A10A and A10B. Lipid-modifying agents include ATC-code C10A. Antihypertensive medication included ATC-codes C03, C07, C08, C09.

PCOS, polycystic ovary syndrome; fp-glucose, fasting plasma glucose; fs-insulin, fasting serum insulin; HOMA-IR, The Homeostatic Model Assessment–insulin resistance; hs-CRP, The high-sensitivity C-reactive protein; MAP, mean arterial pressure.

### Compositional means of the 24-h day

Table 2 displays the compositional means of MVPA, LPA, SB, and sleep for the controls and women with PCOS. In both groups, sleep occupied the largest part of the 24-h cycle, while MVPA occupied the smallest part. A comparison of the compositional means showed that women with PCOS had lower MVPA levels. However, Hotelling’s *T*^2^ test showed no significant difference in the overall 24-h movement composition between the two groups (*P* = 0.192).

**Table 2.** Compositional means of behaviours within 24-h cycle in controls and in women with PCOS.

Movement behaviours	Control (n* *=* *665)	PCOS (n* *=* *192)	Percentage difference (Cl 95%)^a^
MVPA	48.9 (3.0%)	45.1 (2.7%)	–0.095% (–0.192 to –0.001)*
LPA	394.5 (27.2%)	395.8 (27.3%)	0.005% (–0.033 to 0.043)
SB	478.4 (33.3%)	477.1 (33.1%)	–0.004% (–0.036 to 0.029)
Sleep	518.2 (36.6%)	522.0 (36.8%)	0.007% (–0.009 to 0.022)

Compositional means are expressed as minutes and as percentages of a 1440 min (24-h) day.

a Indicates the log ratio difference between controls and women with PCOS converted into percentage. Percentage difference and 95% CIs were determined with bootstrapping.

* 95% CI does not intersect zero, thus MVPA is different between the groups. Negative estimated difference indicates that controls have a greater level of the given component compared to women with PCOS.

PCOS, polycystic ovary syndrome; MVPA, moderate-to-vigorous physical activity; LPA, light physical activity; SB, sedentary behaviour.

### Compositional linear regression models

Table 3 displays the models’ *R*^2^ and *P*-values for the overall 24-h movement composition. In the control group, the associations between the 24-h movement composition and cardiometabolic markers were significant in all models. For women with PCOS, the associations between the 24-h movement composition and waist circumference, triglycerides, fs-insulin, and HOMA-IR were significant in all models, whereas the unadjusted associations with fp-glucose, 2-h glucose, 2-h insulin, hs-CRP, and MAP were not significant. The univariate linear regression model results between cardiometabolic markers and time spent on each behaviour relative to the rest of the 24-h composition are shown in Supplementary Table S2. A curvilinear association between mean sleep duration and 2-h glucose was found in the control group; the results of the stratified models are shown also in Supplementary Table S3.

**Table 3.** Associations between overall 24-h movement composition and cardiometabolic markers in controls and women with PCOS.

	Control			PCOS		
Cardiometabolic markers	*n*	Model *R*^2^	Model *P*	n	Model *R*^2^	Model *P*
**Waist circumference**						
Crude	663	0.12	**<0.001**	192	0.08	**<0.001**
Model 1	614	0.16	**<0.001**	177	0.21	**<0.001**
Model 2	601	0.18	**<0.001**	175	0.22	**<0.001**
**Triglycerides **						
Crude	665	0.08	**<0.001**	192	0.08	**<0.001**
Model 1	616	0.11	**<0.001**	178	0.12	**0.028 **
Model 2	603	0.11	**<0.001**	176	0.12	**0.046**
Model 3	603	0.24	**<0.001**	176	0.29	**<0.001**
**fp-glucose **						
Crude	656	0.05	**<0.001**	186	0.03	0.153
Model 1	607	0.05	**<0.001**	172	0.12	**0.034 **
Model 2	594	0.06	**<0.001**	170	0.13	**0.032**
Model 3	594	0.13	**<0.001**	170	0.35	**<0.001**
**fs-insulin**						
Crude	660	0.10	**<0.001**	187	0.12	**<0.001**
Model 1	611	0.14	**<0.001**	173	0.20	**<0.001**
Model 2	598	0.16	**<0.001**	171	0.20	**<0.001**
Model 3	598	0.35	**<0.001**	171	0.50	**<0.001**
**HOMA-IR**						
Crude	634	0.08	**<0.001**	180	0.08	**0.003**
Model 1	587	0.12	**<0.001**	166	0.16	**0.004 **
Model 2	574	0.14	**<0.001**	164	0.17	**0.005**
Model 3	574	0.35	**<0.001**	164	0.56	**<0.001**
**2-h glucose** * ** **						
Crude	586	0.03	**0.005**	163	0.003	0.928
Model 1	544	0.09	**<0.001**	151	0.07	0.556
Model 2	533	0.10	**<0.001**	150	0.10	0.247
Model 3	533	0.15	**<0.001**	150	0.17	**0.014**
**2-h insulin **						
Crude	584	0.09	**<0.001**	165	0.02	0.462
Model 1	543	0.14	**<0.001**	153	0.08	0.410
Model 2	532	0.14	**<0.001**	152	0.10	0.199
Model 3	532	0.25	**<0.001**	152	0.25	**<0.001**
**hs-CRP**						
Crude	654	0.07	**<0.001**	186	0.03	0.170
Model 1	607	0.08	**<0.001**	173	0.09	0.193
Model 2	594	0.09	**<0.001**	171	0.08	0.289
Model 3	594	0.31	**<0.001**	171	0.29	**<0.001**
**MAP**						
Crude	661	0.03	**<0.001**	191	0.02	0.283
Model 1	613	0.10	**<0.001**	177	0.08	0.278
Model 2	600	0.09	**<0.001**	175	0.09	0.243
Model 3	600	0.23	**<0.001**	175	0.17	**0.002**

The table includes *R*^2^ values and statistical significances for each compositional model created for each cardiometabolic marker (model *P-*value). Statistically significant associations (*P *<* *0.05) are bolded. Model 1 is adjusted for education, marital status, alcohol use, smoking, psychological distress, medication use (blood glucose lowering, lipid modifying, or antihypertensive). Model 2 is further adjusted with the Healthy Diet Index. Model 3 is further adjusted with the Healthy Diet Index and BMI.

* In controls, showed a U-shaped relationship with mean sleep duration (<8.5 h and ≥8.5 h).

PCOS, polycystic ovary syndrome; fp-glucose, fasting plasma glucose; fs-insulin, fasting serum insulin; HOMA-IR, The Homeostatic Model Assessment–insulin resistance; hs-CRP, The high-sensitivity C-reactive protein; MAP, mean arterial pressure.

The adjusted associations between cardiometabolic markers and time spent on MVPA and LPA relative to the rest of the 24-h composition are presented in Table 4. In Models 1 and 2, more time spent daily on MVPA relative to the rest of the behaviours was negatively associated with waist circumference, triglycerides, fs-insulin, and HOMA-IR in both groups. However, unlike women with PCOS, the controls also showed negative associations between MVPA and fp-glucose, 2-h glucose, 2-h insulin, hs-CRP, and MAP when adjusting for the rest of the 24-h movement composition. No significant associations remained after further adjustment for BMI, except regarding 2-h insulin among the controls. The attenuation in effect size after BMI-adjustment was more pronounced in women with PCOS compared to controls. There were no significant MVPA-group interactions.

**Table 4.** Associations between cardiometabolic markers and MVPA and LPA in controls and women with PCOS.

	Control			PCOS			
Cardiometabolic markers	β	95% Cl	*P*-value	β	95% Cl	*P*-value	*P*-value int.^a^
**MVPA**							
**Waist circumference**							
Model 1	–0.19	–0.27 to –0.12	**<0.001**	–0.23	–0.38 to –0.08	**<0.001**	0.471
Model 2	–0.20	–0.28 to –0.12	**<0.001**	–0.22	–0.37 to –0.06	**0.006**	0.558
**Triglycerides**							
Model 1	–0.11	–0.19 to –0.03	**0.009**	–0.23	–0.39 to –0.07	**0.004**	0.271
Model 2	–0.10	–0.18 to –0.02	**0.017**	–0.23	–0.39 to –0.06	**0.007**	0.249
Model 3	–0.01	–0.09 to 0.07	0.754	–0.11	–0.26 to 0.04	0.160	0.391
**fp-glucose**							
Model 1	–0.11	–0.19 to –0.02	**0.013**	–0.02	–0.18 to 0.15	0.851	0.886
Model 2	–0.11	–0.19 to –0.02	**0.014**	–0.001	–0.16 to 0.16	0.998	0.851
Model 3	–0.04	–0.12 to 0.05	0.367	0.14	0.00 to 0.29	0.055	0.556
**fs-insulin**							
Model 1	–0.15	–0.23 to –0.07	**<0.001**	–0.31	–0.46 to –0.15	**<0.001**	0.073
Model 2	–0.15	–0.23 to –0.07	**<0.001**	–0.30	–0.46 to –0.14	**<0.001**	0.072
Model 3	–0.04	–0.11 to 0.04	0.313	–0.12	–0.25 to 0.01	0.068	0.154
**HOMA-IR**							
Model 1	–0.13	–0.21 to –0.05	**0.002**	–0.23	–0.39 to –0.07	**0.006**	0.227
Model 2	–0.13	–0.21 to –0.05	**0.002**	–0.21	–0.38 to –0.05	**0.012**	0.221
Model 3	–0.02	–0.09 to 0.06	0.635	–0.004	–0.13 to 0.12	0.956	0.555
**2-h glucose** *							
Model 1	–0.11	–0.20 to –0.03	**0.012**	–0.06	–0.24 to 0.12	0.492	0.595
Model 2	–0.11	–0.20 to –0.02	**0.014**	–0.04	–0.22 to 0.14	0.671	0.542
Model 3	–0.06	–0.15 to 0.03	0.189	0.04	–0.14 to 0.22	0.668	0.382
**2-h insulin**							
Model 1	–0.20	–0.28 to –0.11	**<0.001**	–0.16	–0.34 to 0.02	0.081	0.380
Model 2	–0.20	–0.28 to –0.11	**<0.001**	–0.14	–0.31 to 0.04	0.133	0.340
Model 3	–0.12	–0.21 to –0.04	**0.003**	–0.03	–0.19 to 0.14	0.753	0.162
**hs-CRP**							
Model 1	–0.18	–0.26 to –0.09	**<0.001**	–0.22	–0.38 to –0.06	**0.009**	0.771
Model 2	–0.16	–0.25 to –0.08	**<0.001**	–0.22	–0.39 to –0.05	**0.010**	0.818
Model 3	–0.05	–0.13 to 0.02	0.146	–0.08	–0.23 to 0.07	0.306	0.335
**MAP**							
Model 1	–0.11	–0.20 to –0.03	**0.006**	–0.09	–0.26 to 0.07	0.264	0.988
Model 2	–0.10	–0.18 to –0.02	**0.016**	–0.08	–0.24 to 0.09	0.343	0.971
Model 3	–0.006	–0.08 to 0.07	0.871	–0.007	–0.16 to 0.17	0.932	0.713
**LPA**							
**Waist circumference**							
Model 1	–0.11	–0.21 to –0.02	**0.019**	–0.05	–0.23 to 0.13	0.561	0.544
Model 2	–0.14	–0.23 to –0.04	**0.006**	–0.05	–0.23 to 0.13	0.593	0.387
**Triglycerides**							
Model 1	–0.20	–0.30 to –0.11	**<0.001**	–0.14	–0.33 to 0.05	0.156	0.451
Model 2	–0.21	–0.31 to –0.11	**<0.001**	–0.14	–0.33 to 0.05	0.150	0.450
Model 3	–0.16	–0.26 to –0.07	**<0.001**	–0.14	–0.31 to 0.04	0.125	0.758
**fp-glucose**							
Model 1	–0.13	–0.23 to –0.02	**0.016** ^#^	–0.15	–0.34 to 0.05	0.146	0.886
Model 2	–0.13	–0.23 to –0.03	**0.013^#^**	–0.14	–0.34 to 0.06	0.156	0.884
Model 3	–0.10	–0.20 to 0.00	0.058	–0.14	–0.31 to 0.03	0.112	0.756
**fs-insulin**							
Model 1	–0.15	–0.25 to –0.06	**0.002**	–0.14	–0.32 to 0.05	0.152	0.682
Model 2	–0.17	–0.26 to –0.07	**<0.001**	–0.14	–0.32 to 0.05	0.158	0.532
Model 3	–0.11	–0.20 to –0.03	**0.011**	–0.16	–0.31 to –0.01	**0.037** ^#^	0.751
**HOMA-IR**							
Model 1	–0.16	–0.26 to –0.06	**0.002**	–0.14	–0.33 to 0.06	0.163	0.627
Model 2	–0.17	–0.28 to –0.07	**<0.001**	–0.14	–0.33 to 0.06	0.172	0.483
Model 3	–0.12	–0.21 to –0.03	**0.009**	–0.16	–0.30 to –0.02	**0.030** ^#^	0.819
**2-h glucose** *							
Model 1	–0.03	–0.16 to 0.10	0.659	0.03	–0.18 to 0.25	0.758	0.523
Model 2	–0.02	–0.13 to 0.08	0.650	0.02	–0.19 to 0.24	0.838	0.534
Model 3	–0.001	–0.10 to 0.10	0.996	0.008	–0.20 to 0.20	0.936	0.845
**2-h insulin**							
Model 1	–0.11	–0.21 to –0.01	**0.035**	0.08	–0.14 to 0.29	0.490	0.132
Model 2	–0.11	–0.21 to –0.01	**0.034**	0.06	–0.15 to 0.28	0.557	0.130
Model 3	–0.07	–0.17 to 0.02	0.131	0.04	–0.16 to 0.24	0.675	0.332
**hs-CRP**							
Model 1	–0.04	–0.14 to 0.06	0.416	0.14	–0.06 to 0.34	0.166	0.158
Model 2	–0.04	–0.14 to 0.06	0.423	0.15	–0.05 to 0.35	0.153	0.156
Model 3	0.01	–0.07 to 0.10	0.755	0.15	–0.03 to 0.32	0.108	0.294
**MAP**							
Model 1	–0.03	–0.13 to 0.07	0.552	0.09	–0.10 to 0.29	0.357	0.062
Model 2	–0.03	–0.13 to 0.07	0.613	0.07	–0.12 to 0.27	0.459	0.101
Model 3	0.02	–0.08 to 0.11	0.707	0.08	–0.11 to 0.27	0.407	0.186

The associations between each movement behaviour and cardiometabolic marker are expressed in relation to the rest of the 24-h movement composition. Standardized beta coefficients (β) are presented with 95% CI. Statistically significant associations (*P *<* *0.05) are bolded.

a *P*-value interaction indicates whether the association between movement behaviour and cardiometabolic marker is statistically different between controls and women with PCOS. Model 1 is adjusted for education, marital status, alcohol use, smoking, psychological distress, medication use (blood glucose lowering, lipid modifying, or antihypertensive). Model 2 is further adjusted with the Healthy Diet Index. Model 3 is further adjusted with the Healthy Diet Index and BMI.

* In controls, showed a U-shaped relationship with mean sleep duration (<8.5 h and ≥8.5 h).

# The variable did not show a statistically significant association in the univariate analysis.

PCOS, polycystic ovary syndrome; fp-glucose, fasting plasma glucose; fs-insulin, fasting serum insulin; HOMA-IR, The Homeostatic Model Assessment–insulin resistance; hs-CRP, The high-sensitivity C-reactive protein; MAP, mean arterial pressure; MVPA, moderate-to-vigorous physical activity; LPA, light physical activity.

In controls, but not in women with PCOS, more time spent daily on LPA relative to the rest of the behaviours was negatively associated with waist circumference, triglycerides, fp-glucose, fs-insulin, HOMA-IR, and 2-h insulin in Models 1 and 2. In BMI-adjusted models, more LPA relative to the rest of the behaviours was negatively associated with fs-insulin and HOMA-IR in both groups. The attenuation in effect size after BMI-adjustment was less pronounced in women with PCOS compared to controls. There were no significant LPA-group interactions.

The adjusted associations between cardiometabolic markers and time spent on SB and sleep relative to the rest of the 24-h composition are presented in Table 5. In Models 1 and 2, more time spent daily on SB relative to the rest of the behaviours was positively associated with waist circumference, fs-insulin, HOMA-IR, 2-h insulin, and MAP values in the control group, whereas no significant associations were observed for women with PCOS. None of the associations were significant after further adjustment for BMI. There were no significant SB-group interactions. In both groups, more time spent daily on sleep relative to the rest of the behaviours was not associated with any cardiometabolic markers in the unadjusted model (Supplementary Table S2). However, in the adjusted models, more sleep relative to the rest of the behaviours was positively associated with triglycerides in the control group and with fs-insulin among women with PCOS. Additionally, in the BMI-adjusted model, more sleep relative to the rest of the behaviours was positively associated with HOMA-IR among women with PCOS. There were no significant sleep-group interactions.

**Table 5.** Associations between cardiometabolic markers, SB and sleep in controls and women with PCOS.

	Control			PCOS			
Cardiometabolic markers	β	95% Cl	*P*-value	β	95% Cl	*P*-value	*P*-value int.^a^
**SB**							
**Waist circumference**							
Model 1	0.23	0.11 to 0.35	**<0.001**	0.09	–0.15 to 0.33	0.454	0.288
Model 2	0.25	0.13 to 0.37	**<0.001**	0.11	–0.13 to 0.36	0.364	0.278
**Triglycerides**							
Model 1	0.09	–0.03 to 0.22	0.139	0.001	–0.25 to 0.25	0.993	0.122
Model 2	0.12	–0.01 to 0.24	0.063	–0.002	–0.26 to 0.26	0.988	0.374
Model 3	0.01	–0.11 to 0.13	0.872	–0.05	–0.28 to 0.19	0.697	0.703
**fp-glucose**							
Model 1	0.07	–0.06 to 0.20	0.303	0.05	–0.20 to 0.31	0.682	0.598
Model 2	0.10	–0.03 to 0.23	0.145	0.07	–0.20 to 0.33	0.623	0.698
Model 3	0.02	–0.11 to 0.14	0.812	–0.003	–0.23 to 0.23	0.979	0.429
**fs-insulin**							
Model 1	0.17	0.04 to 0.29	**0.008**	–0.09	–0.34 to 0.16	0.477	0.082
Model 2	0.20	0.08 to 0.32	**0.002**	–0.08	–0.34 to 0.18	0.537	0.072
Model 3	0.06	–0.05 to 0.17	0.248	–0.16	–0.37 to 0.04	0.112	0.147
**HOMA-IR**							
Model 1	0.14	0.01 to 0.27	**0.031**	–0.05	–0.31 to 0.21	0.710	0.241
Model 2	0.17	0.05 to 0.30	**0.007**	–0.04	–0.30 to 0.23	0.791	0.214
Model 3	0.04	–0.07 to 0.15	0.485	–0.14	–0.33 to 0.06	0.170	0.360
2-h glucose^*^							
Model 1	0.15	–0.02 to 0.32	0.077	–0.05	–0.33 to 0.24	0.743	0.394
Model 2	0.17	0.04 to 0.31	**0.009**	–0.03	–0.32 to 0.25	0.817	0.363
Model 3	0.10	–0.03 to 0.23	0.143	–0.03	–0.31 to 0.24	0.810	0.706
**2-h insulin**							
Model 1	0.16	0.03 to 0.29	**0.014**	–0.14	–0.42 to 0.14	0.320	0.160
Model 2	0.18	0.05 to 0.31	**0.007**	–0.13	–0.41 to 0.15	0.357	0.134
Model 3	0.07	–0.06 to 0.19	0.283	–0.13	–0.39 to 0.13	0.311	0.419
**hs-CRP**							
Model 1	0.13	0.00 to 0.26	**0.042**	0.11	–0.15 to 0.36	0.413	0.740
Model 2	0.14	0.02 to 0.27	**0.028**	0.12	–0.15 to 0.39	0.371	0.781
Model 3	–0.002	–0.11 to 0.11	0.979	0.10	–0.13 to 0.34	0.396	0.472
**MAP**							
Model 1	0.16	0.03 to 0.28	**0.016**	0.18	–0.08 to 0.43	0.178	0.785
Model 2	0.16	0.03 to 0.28	**0.015**	0.15	–0.12 to 0.41	0.279	0.949
Model 3	0.04	–0.08 to 0.16	0.507	0.12	–0.14 to 0.37	0.370	0.525
**Sleep**							
**Waist circumference**							
Model 1	0.01	–0.11 to 0.13	0.840	0.08	–0.16 to 0.31	0.513	0.606
Model 2	0.02	–0.10 to 0.13	0.791	0.05	–0.19 to 0.29	0.655	0.980
**Triglycerides**							
Model 1	0.13	0.01 to 0.25	**0.036** ^#^	0.20	–0.04 to 0.45	0.104	0.784
Model 2	0.11	–0.01 to 0.23	0.069	0.21	–0.05 to 0.46	0.113	0.673
Model 3	0.12	0.00 to 0.23	**0.042** ^#^	0.18	–0.05 to 0.41	0.119	0.778
**fp-glucose**							
Model 1	0.09	–0.04 to 0.22	0.164	0.09	–0.16 to 0.35	0.485	0.677
Model 2	0.07	–0.05 to 0.20	0.247	0.07	–0.19 to 0.34	0.582	0.700
Model 3	0.07	–0.05 to 0.20	0.226	0.06	–0.17 to 0.29	0.627	0.637
**fs-insulin**							
Model 1	0.06	–0.06 to 0.18	0.314	0.29	0.05 to 0.53	**0.019** ^#^	0.182
Model 2	0.05	–0.07 to 0.17	0.424	0.28	0.03 to 0.53	**0.029** ^#^	0.213
Model 3	0.05	–0.05 to 0.16	0.327	0.28	0.08 to 0.48	**0.006** ^#^	0.137
**HOMA-IR**							
Model 1	0.08	–0.05 to 0.20	0.213	0.23	–0.02 to 0.48	0.067	0.435
Model 2	0.06	–0.06 to 0.19	0.307	0.22	–0.04 to 0.48	0.099	0.282
Model 3	0.07	–0.04 to 0.17	0.227	0.22	0.03 to 0.40	**0.026** ^#^	0.379
2-h glucose^*^							
Model 1	–0.03	–0.21 to 0.15	0.731	0.03	–0.25 to 0.31	0.847	0.977
Model 2	–0.05	–0.18 to 0.07	0.427	0.02	–0.26 to 0.30	0.897	0.953
Model 3	–0.04	–0.16 to 0.08	0.527	0.009	–0.27 to 0.27	0.995	0.923
**2-h insulin**							
Model 1	0.06	–0.07 to 0.18	0.378	0.09	–0.19 to 0.37	0.514	0.817
Model 2	0.04	–0.08 to 0.17	0.479	0.09	–0.19 to 0.37	0.540	0.847
Model 3	0.06	–0.05 to 0.18	0.302	0.06	–0.19 to 0.32	0.633	0.672
**hs-CRP**							
Model 1	0.01	–0.11 to 0.14	0.817	–0.09	–0.35 to 0.16	0.464	0.477
Model 2	0.002	–0.12 to 0.13	0.977	–0.11	–0.37 to 0.16	0.418	0.468
Model 3	0.01	–0.09 to 0.12	0.806	–0.15	–0.39 to 0.08	0.201	0.180
**MAP**							
Model 1	–0.03	–0.16 to 0.09	0.592	–0.15	–0.40 to 0.10	0.247	0.192
Model 2	–0.04	–0.17 to 0.08	0.486	–0.12	–0.38 to 0.14	0.360	0.314
Model 3	–0.04	–0.15 to 0.08	0.524	–0.14	–0.39 to 0.11	0.267	0.195

The associations between each movement behaviour and cardiometabolic marker are expressed in relation to the rest of the 24-h movement composition. Standardized beta coefficients (β) are presented with 95% CI. Statistically significant associations (*P *<* *0.05) are bolded.

a *P*-value interaction indicates whether the association between movement behaviour and cardiometabolic marker is statistically different between controls and women with PCOS. Models are adjusted for education, marital status, alcohol use, smoking, psychological distress, and medication use (blood glucose lowering, lipid modifying, or antihypertensive). Model 2 is further adjusted with the Healthy Diet Index. Model 3 is further adjusted with the Healthy Diet Index and BMI.

* In controls, showed a U-shaped relationship with mean sleep duration (<8.5 h and ≥8.5 h).

# The variable did not show a statistically significant association in the univariate analysis.

PCOS, polycystic ovary syndrome; fp-glucose, fasting plasma glucose; fs-insulin, fasting serum insulin; HOMA-IR, The Homeostatic Model Assessment–insulin resistance; hs-CRP, The high-sensitivity C-reactive protein; MAP, mean arterial pressure; SB, sedentary behaviour.

### Sub-group analysis

The compositional means of MVPA, LPA, SB, and sleep for PCOS-phenotypes are shown in Supplementary Table S4; there were no significant differences in the overall 24-h movement composition between the phenotypes. The univariate associations between the 24-h movement composition and cardiometabolic markers in PCOS-phenotypes are shown in Supplementary Table S5. Among the A + B-phenotype, there were no significant associations between the overall 24-h movement composition and cardiometabolic markers, whereas the C-phenotype showed a significant association between the 24-h movement composition and triglycerides. However, when the A + B and C-phenotypes were combined as the ‘HA-phenotype’, significant associations emerged between the overall 24-h movement composition and waist circumference, triglycerides, and fs-insulin. Among the D-phenotype (‘non-HA-phenotype’), there were significant associations between the overall 24-h movement composition and waist circumference, triglycerides, fs-insulin, HOMA-IR, and 2-h insulin.

### Isotemporal reallocation model

**Figure 1.**
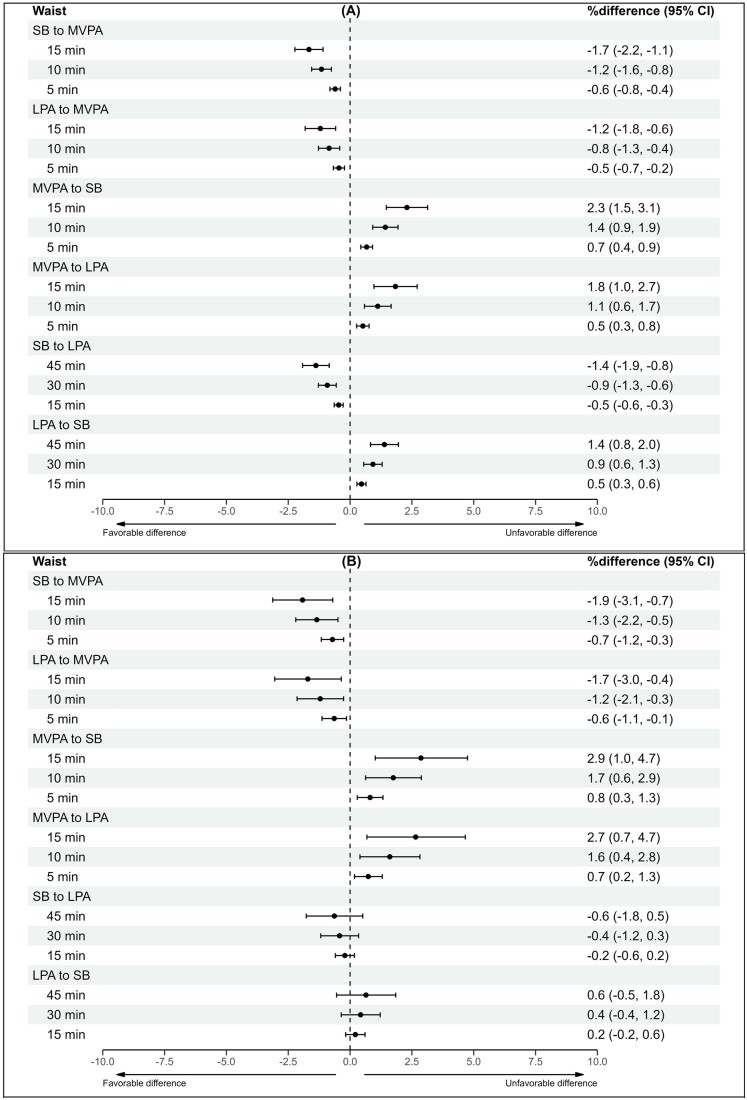
**The differences in waist circumference estimates with varying 24-h composition**. (**A**) Controls and (**B**) women with PCOS. Estimates are expressed as percentage differences (95% CI) when varying totals of MVPA, LPA, and SB in a pairwise manner within the 24-h movement composition. Adjusted for education, marital status, alcohol use, smoking, psychological distress, and medication use (blood glucose lowering, lipid modifying, or antihypertensive). MVPA, moderate-to-vigorous physical activity; LPA, light physical activity; SB: sedentary behaviour.

**Figure 2.**
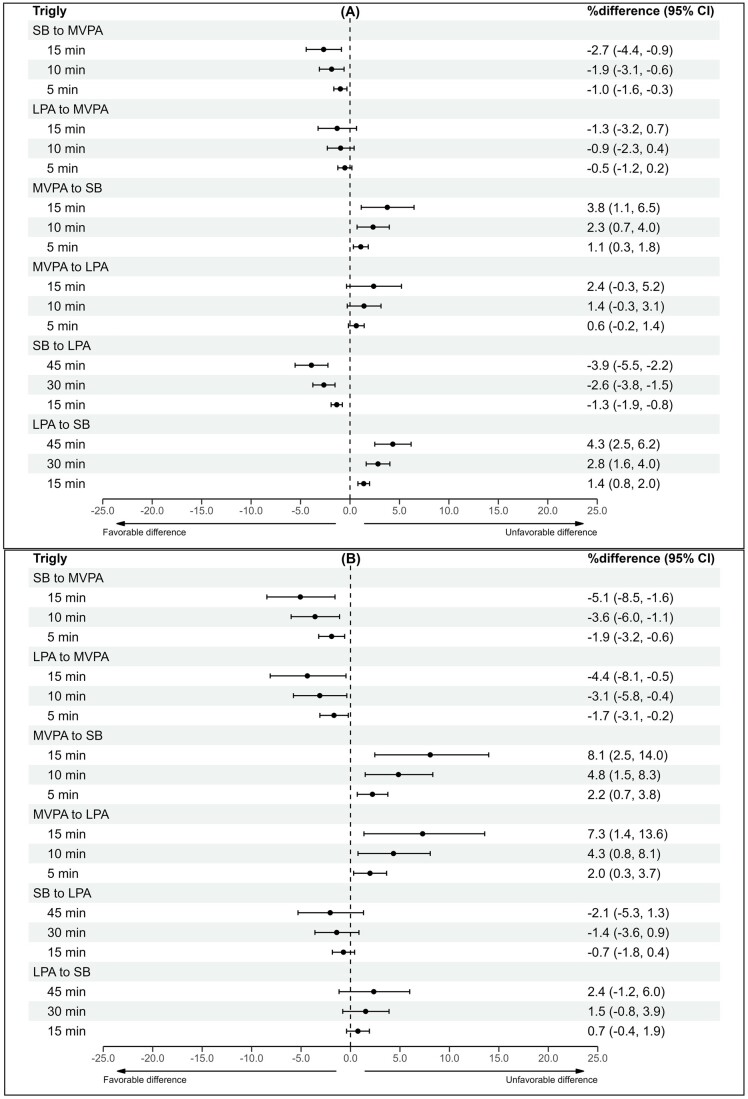
**The differences in triglyceride estimates with varying 24-h composition**. (**A**) Controls and (**B**) women with PCOS. Estimates are expressed as percentage differences (95% CI) when varying totals of MVPA, LPA, and SB in a pairwise manner within the 24-h movement composition. Adjusted for education, marital status, alcohol use, smoking, psychological distress, and medication use (blood glucose lowering, lipid modifying, or antihypertensive). MVPA, moderate-to-vigorous physical activity; LPA, light physical activity; SB, sedentary behaviour; trigly, triglycerides.

**Figure 3.**
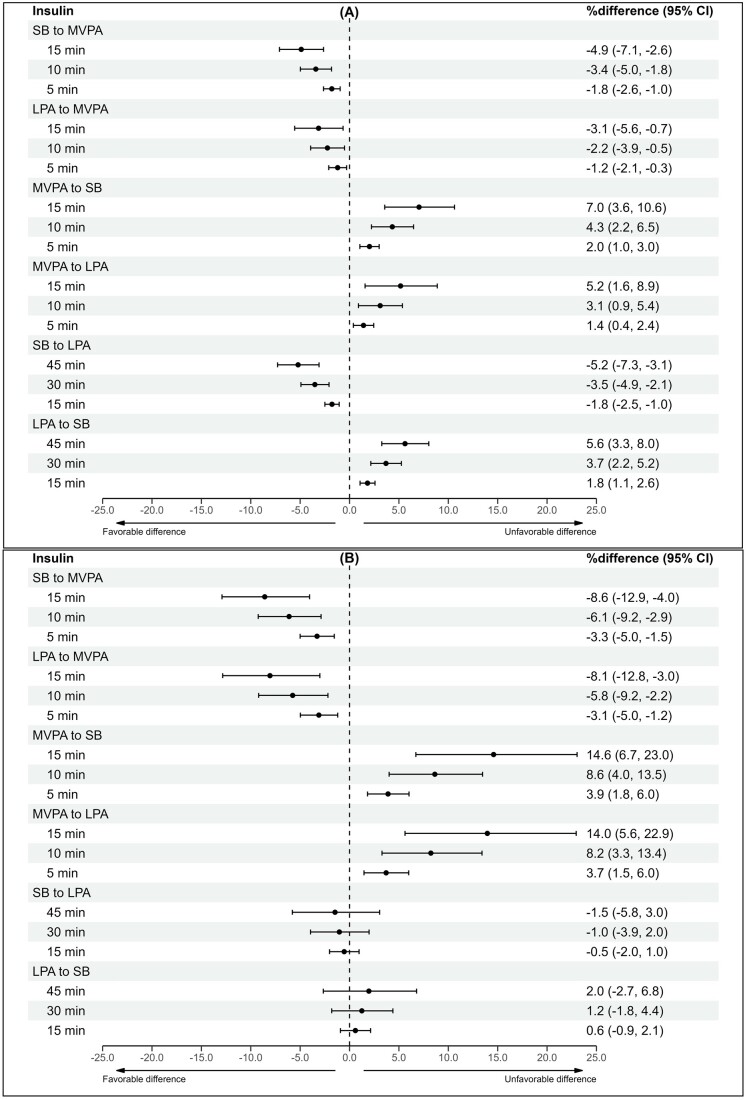
**The differences in fs-insulin estimates with varying 24-h composition**. (**A**) Controls and (**B**) women with PCOS. Estimates are expressed as percentage differences (95% CI) when varying totals of MVPA, LPA, and SB in a pairwise manner within the 24-h movement composition. Adjusted for education, marital status, alcohol use, smoking, psychological distress, and medication use (blood glucose lowering, lipid modifying, or antihypertensive). MVPA, moderate-to-vigorous physical activity; LPA, light physical activity; SB, sedentary behaviour; fs-insulin, fasting serum insulin.

**Figure 4.**
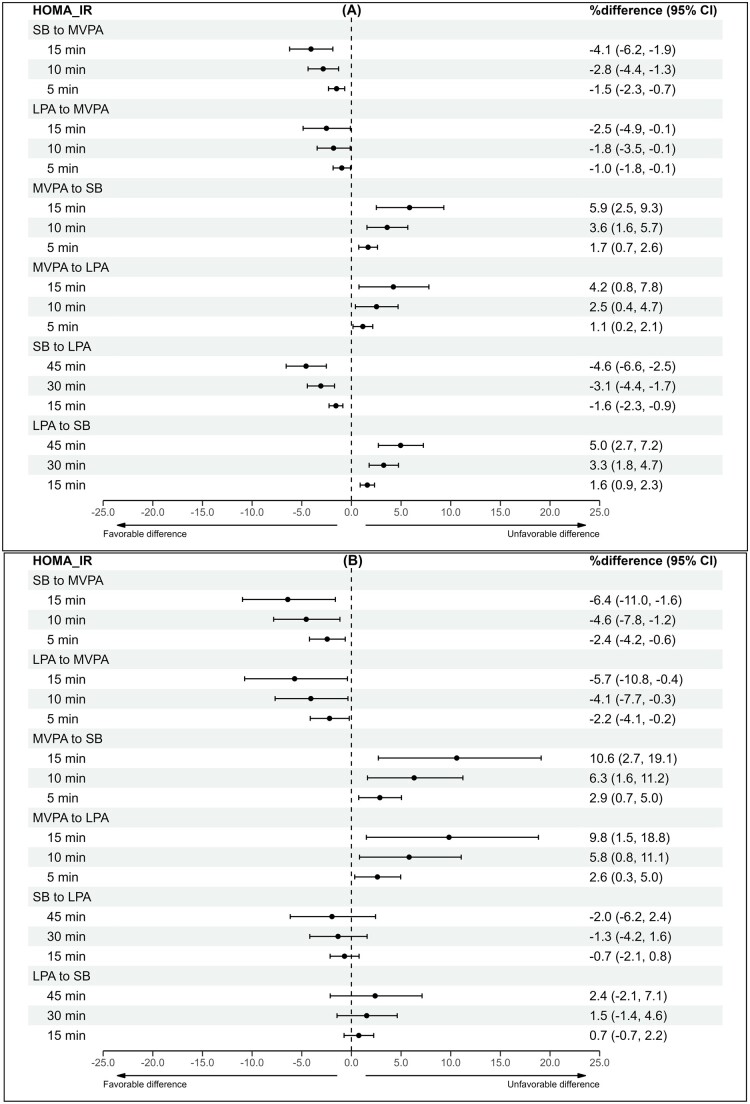
**The difference in HOMA-IR estimates with varying 24-h composition**. (**A**) Controls and (**B**) women with PCOS. Estimates are expressed as percentage differences (95% CI) when varying totals of MVPA, LPA, and SB in a pairwise manner within the 24-h movement composition. Adjusted for education, marital status, alcohol use, smoking, psychological distress, and medication use (blood glucose lowering, lipid modifying, or antihypertensive). MVPA, moderate-to-vigorous physical activity; LPA, light physical activity; SB, sedentary behaviour; HOMA-IR, The Homeostatic Model Assessment–insulin resistance.

Time reallocations were performed for waist circumference, triglycerides, fs-insulin, and HOMA-IR due to their significant associations with the overall 24-h movement composition in univariate analyses among both groups. The results of the time reallocations between MVPA, LPA, and SB are presented in Figs 1, 2, 3, and 4. In both groups, increasing MVPA at the expense of any other behaviour was associated with favourable differences in cardiometabolic marker estimates in a dose-responsive manner, although the beneficial associations were less apparent in the control group when LPA time was reallocated to MVPA. In the control group, increasing MVPA by 15 min while decreasing SB by 15 min was associated with lower waist circumference (−1.7%Δ [95% CI −2.2 to −1.1]), triglyceride (−2.7%Δ [95% CI −4.4 to −0.9]), fs-insulin (−4.9%Δ [95% CI −7.1 to −2.6]), and HOMA-IR (−4.1%Δ [95% CI −6.2 to −1.9) values. For women with PCOS, the estimated percentage differences were greater. Reallocating 15 min from SB to MVPA was associated with the following beneficial difference estimates: waist circumference (−1.9%Δ [95% CI −3.1 to −0.7]), triglycerides (−5.1%Δ [95% CI −8.5 to −1.6]), fs-insulin (−8.6%Δ [95% CI −12.9 to −4.0]), and HOMA-IR (−6.4%Δ [95% CI −11.0 to −1.6]).

When time was reallocated from MVPA to any other behaviour, the differences in cardiometabolic marker estimates were considerably greater than when time was reallocated from any behaviour to MVPA. In the control group, reducing MVPA by 15 min while increasing SB by 15 min was positively associated with waist circumference (2.3%Δ [95% CI 1.5–3.1]), triglyceride (3.8%Δ [95% CI 1.1–6.5]), fs-insulin (7.0%Δ [95% CI 3.6–10.6]), and HOMA-IR (5.9%Δ [95% CI 2.5–9.3]) values. In women with PCOS, the percentage differences were greater in the following cardiometabolic marker estimates: waist circumference (2.9%Δ [95% CI 1.0–4.7]), triglycerides (8.1%Δ [95% CI 2.5–14.0]), fs-insulin (14.6%Δ [95% CI 6.7–23.0]), and HOMA-IR (10.6%Δ [95% CI 2.7–19.1]).

Reallocating time between LPA and SB was significantly associated with differences in cardiometabolic marker estimates for the controls but not for women with PCOS. In the control group, decreasing SB by 15 min while increasing LPA by 15 min was associated with lower waist circumference (−0.5%Δ [95% CI −0.6 to −0.3]), triglyceride (−1.3%Δ [95% CI −1.9 to −0.8]), fs-insulin (−1.8%Δ [95% CI −2.5 to −1.0]), and HOMA-IR (−1.6%Δ [95% CI −2.3 to −0.9]) values. For women with PCOS, the same time reallocation resulted in no significant associations. In the controls, reallocating 15 min of LPA to SB was significantly associated with higher waist circumference (0.5%Δ [95% CI 0.3–0.6]), triglyceride (1.4%Δ [95% CI 0.8–2.0]), fs-insulin (1.8%Δ [95% CI 1.1–2.6]), and HOMA-IR (1.6%Δ [95% CI 0.9–2.3]) values. The same time reallocation showed no significant associations for women with PCOS.

The time reallocations from and to sleep are presented in Supplementary Figs S2, S3, S4, and S5. In the control group, reallocating 15 min from sleep to LPA was associated with lower triglyceride (−1.7%Δ [95% CI −2.7 to −0.6]), fs-insulin (−1.4%Δ [95% CI −2.7 to −0.1]), and HOMA-IR (−1.4%Δ [95% CI −2.6 to −0.2]) levels. For women with PCOS, this time reallocation was significantly associated only with fs-insulin (−2.9%Δ [95% CI −5.5 to −0.2]). For controls, reallocating 15 min from LPA to sleep was significantly associated with higher triglyceride (1.7%Δ [95% CI 0.7–2.7]), fs-insulin (1.4%Δ [95% CI 0.1–2.8]), and HOMA-IR (1.5%Δ [95% CI 0.2–2.7]) levels, whereas for women with PCOS, it was significantly associated only with fs-insulin (2.9%Δ [95% CI 0.2–5.8]).

## Discussion

To our knowledge, this is the first study to use CoDA to examine the codependent relationships between 24-h movement behaviours and cardiometabolic markers in women with and without PCOS. The 24-h movement composition was associated with waist circumference, triglycerides, fs-insulin, and HOMA-IR in both groups. The favourable differences in cardiometabolic markers when decreasing SB time while increasing MVPA time were consistently greater among women with PCOS, potentially indicating a stronger association to MVPA in this population. However, unlike the controls, women with PCOS showed no significant differences in cardiometabolic markers when reallocating SB time to LPA, which may reflect a weaker association to LPA among affected women. Given the increasing prevalence of activity recommendations including phrases such as ‘every move counts’ (Ekelund *et al.*, 2019b; Teede *et al.*, 2023), it is important to be aware that replacing SB with LPA alone may not be sufficient to mitigate cardiometabolic risk in all subpopulations, despite the well-known health benefits of physical activity. Our findings warrant further research and underline the importance of developing tailored activity guidelines for women with PCOS.

The overall 24-h movement composition did not differ significantly between women with and without PCOS, although a small difference (0.1%; ∼1.4 min) was observed in MVPA relative to the rest of the behaviours. Prior observational studies using questionnaire data have reported lower physical activity levels and longer sedentary times among women with than without PCOS (Tay *et al.*, 2020; Kazemi *et al.*, 2022). However, in line with our findings, Lin *et al.* (2019) reported no significant differences between women with and without PCOS in terms of physical activity duration, type, or intensity measured using both questionnaires and waist-worn accelerometers, although they did not use CoDA to analyse their results. We also found no difference in sleep duration between women with and without PCOS when adjusting for the rest of the composition. While this is line with a prior study using self-reported sleep data (Bennett *et al.*, 2022), objectively measured total sleep duration and sleep efficiency have been found to be lower in women with PCOS than in women without the syndrome (Oberg *et al.*, 2023).

While we observed no significant movement behaviour-group interactions, which may suggest that there are no differential relationships between movement behaviours and cardiometabolic markers in women with and without PCOS, notable disparities emerged. Among women with PCOS, we found that more MVPA relative to other behaviours was significantly associated with lower waist circumference, triglyceride, fs-insulin, and HOMA-IR values, while among the controls, in addition to these cardiometabolic markers, we observed beneficial associations with fp-glucose, 2-h glucose, 2-h insulin, hs-CRP, and MAP. Notably, in both groups, most of these associations became non-significant after BMI adjustment, indicating that weight change had a significant role in mediating the relationship between MVPA and cardiometabolic health. Adjusting for dietary quality did not change the results. While the lack of significant associations with post-OGTT variables in women with PCOS may be due to smaller sample size and inadequate power, the result could also stem from women with PCOS displaying impaired glucose uptake and insulin signalling in skeletal muscle due to fibrosis and/or metabolic inflexibility (Hansen *et al.*, 2020; Stepto *et al.*, 2020; McDonnell *et al.*, 2022). On the other hand, the lack of significant associations with blood pressure and inflammation may be related to altered cardiac autonomic (Ollila *et al.*, 2019) and endothelial function (Sprung *et al.*, 2013; Lambert *et al.*, 2015), although other studies have reported beneficial adaptations in women with PCOS after exercise training (Giallauria *et al.*, 2008; Benham *et al.*, 2018).

The favourable differences in waist circumference, triglyceride, fs-insulin, and HOMA-IR values when decreasing SB time while increasing MVPA time were consistently greater among women with PCOS. This aligns with the study of Harrison *et al.* (2012), who found a significant association between improved fitness and improved insulin sensitivity in the PCOS, but not in the non-PCOS control group, after an intensified exercise intervention. Indeed, women with PCOS may require intensive physical activity to elicit improvements in metabolic health. Non-compositional evidence obtained from both observational settings (Greenwood *et al.*, 2016) and exercise interventions (Patten *et al.*, 2020) suggests that vigorous physical activity is superior to moderate-intensity activity in terms of reducing the risk of metabolic syndrome in women with PCOS. However, the 2023 evidence-based guidelines for women with PCOS note that there is still overall a lack of evidence suggesting that any one type or intensity of exercise is better than another (Teede *et al.*, 2023).

The 2023 guidelines also suggest that replacing SB with physical activity of any intensity, including LPA, provides health benefits (Teede *et al.*, 2023). However, in the present study, among women with PCOS, reducing SB was associated with improved cardiometabolic markers only when it was replaced with MVPA, not LPA. This aligns with prior evidence indicating that among women with PCOS, reducing SB time while increasing LPA time is not sufficient to alter femoral artery flow-mediated dilation, a marker of endothelial function used as a prognostic marker for cardiovascular disease risk (Taylor *et al.*, 2021). Indeed, the difference in cardiometabolic markers to varying levels of LPA appeared to be blunted in women with PCOS. Unlike the controls, reducing LPA while increasing SB was not associated with unfavourable differences in cardiometabolic health in this group. This finding is in contrast with Brakenridge *et al.*’s (2021) study, which reported that compositions with shorter LPA times and equivalently longer SB times showed detrimental associations only for individuals at a higher risk of diabetes, a group comparable to women with PCOS. Interestingly, a meta-analysis reported that the effects of prolonged sitting on vascular function were weaker among individuals with metabolic disturbances than among healthy individuals, suggesting that SB had less harmful consequences for the former (Taylor *et al.*, 2022). While in the present study, the main findings suggest that replacing SB time only with MVPA is beneficial for women with PCOS, the secondary analysis with BMI-adjustment indicated favourable associations between LPA and insulin sensitivity among women with PCOS. Thus, further studies are needed to determine whether reducing SB while increasing LPA time could suffice as a weight-loss independent method for managing cardiometabolic risk in this population.

This study provides encouraging, albeit theoretical, evidence that even a minor increase of 5–15 min in MVPA, such as brisk walking, at the expense of other behaviours may be associated with improved cardiometabolic health for women with PCOS. In line with this finding, for inactive individuals among the general population, as little as 5 min of brisk walking per day has been suggested to make a clinically important difference in obtaining health benefits (Rowlands *et al.*, 2021). Importantly, our theoretical estimations for MVPA also appear to have clinical significance in terms of differences in adiposity. A 3% reduction in waist circumference, equivalent to 1.8–4.1 cm, is considered clinically relevant (Verweij *et al.*, 2013). In our study, increasing MVPA by 15 min at the expense of SB was associated with a 1.9% (∼1.7 cm) smaller waist circumference among women with PCOS. Extrapolating the time increase to 30 min would be associated with a waist circumference reduction of 3.4% or 3 cm. In comparison, metformin, a medication commonly used to improve insulin sensitivity in women with PCOS, has been associated with an average of 1.4 cm greater decrease in waist circumference compared to control treatments (Guan *et al.*, 2020), while no differences have been found in fs-insulin, triglycerides, and waist–hip ratio when specifically compared to lifestyle treatment (Melin *et al.*, 2023). Increasing MVPA at the expense of other behaviours could be an addition, or potentially an alternative, to medication-based treatment.

In this study, we observed the greatest estimated differences in cardiometabolic markers when reallocating MVPA time to any other behaviour. These unfavourable differences were pronounced in women with PCOS, indicating that they may be more vulnerable in terms of lifestyle behaviours. This is consistent with a previous study reporting that women with PCOS, especially those who did not adhere to physical activity guidelines, exhibited greater longitudinal weight gain than women without PCOS (Awoke *et al.*, 2021). In women with PCOS, the wide CIs in the differences estimated when reducing MVPA in favour of any other behaviour could be explained by the smaller sample size of this group or may indicate variation within the affected women due to a range of PCOS-phenotypes (Dapas *et al.*, 2020). Indeed, our subgroup analysis indicated weaker associations between the 24-h movement composition and cardiometabolic markers among the phenotypes with HA compared to the phenotype without HA, possibly due to more severe metabolic deterioration. Future studies should investigate how cardiometabolic marker responses vary across PCOS subgroups after lifestyle modifications with large enough sample sizes to reliably detect potential differences. Nevertheless, our findings highlight the importance of maintaining or increasing MVPA levels for women with PCOS. For this population, it is essential to address barriers, including fatigue, body image concerns, and psychological distress, and to provide practical support to create, achieve, and sustain realistic physical activity goals (Banting *et al.*, 2014; Colombo *et al.*, 2023).

The main strengths of this study were the device-based MVPA, LPA, and SB measurements in a real-life context with a relatively large, unselected population-based sample size and the analysis of compositional data, which allowed us to examine the entire spectrum of 24-h movement behaviours. Moreover, we identified women with PCOS in accordance with the 2023 International Evidence-based Guideline (Teede *et al.*, 2023). Furthermore, we assessed a wide range of cardiometabolic markers, including fasting and post-OGTTs, while adjusting for multiple confounding factors including dietary quality. Limitations of this study include the self-reported sleeping times, the exclusively Caucasian race of the participants, and the difference in the group sample sizes. Furthermore, the cross-sectional analyses precluded causal interferences. The time gap between definition of the study population at age 31 and conduction of the activity measurements at age 46 could be considered as a limitation; however, it is important to note that the features and diagnosis of PCOS endure beyond the reproductive age. We used AMH as a surrogate for PCOM because in a large population-based cohort study including thousands of participants, it was not feasible to perform a transvaginal ultrasound. The use of the diagnostic criteria according to the 2023 International Evidence-based Guideline for the assessment and management of PCOS in this study may compromise the comparison with previous studies that have used older criteria for PCOS identification. We estimated insulin resistance based on HOMA-IR, as it was not feasible to perform hyperinsulinemic-euglycemic clamps on so many individuals. However, this is a reasonable surrogate for large-scale epidemiological studies. Finally, our findings need to be explored in broader populations, such as among women receiving fertility treatments.

## Conclusions

Increasing MVPA by 5–15 min at the expense of any other behaviour was associated with more favourable waist circumference, triglyceride, fs-insulin, and HOMA-IR values in both women with and without PCOS. Notably, reducing SB while increasing LPA by 15–45 min was associated with beneficial differences in cardiometabolic markers among women without PCOS but not among women with PCOS. These findings suggest that women with PCOS should be preferentially targeted with interventions involving physical activity of at least moderate intensity to improve their cardiometabolic health. Further research is needed to determine whether generic recommendations, such as ‘any activity is good’, are applicable to this population.

## Supplementary Material

deae232_Supplementary_Figure_S1

deae232_Supplementary_Figure_S2

deae232_Supplementary_Figure_S3

deae232_Supplementary_Figure_S4

deae232_Supplementary_Figure_S5

deae232_Supplementary_Table_S1

deae232_Supplementary_Table_S2

deae232_Supplementary_Table_S3

deae232_Supplementary_Table_S4

deae232_Supplementary_Table_S5

## Data Availability

The data of the current study are not publicly available but are available on reasonable request. The cohort centre grants the final study permit.
