# Supplementary figures and images for: 24-hour movement behaviours and cardiometabolic markers in women with polycystic ovary syndrome (PCOS): a compositional data analysis
Source: Hum Reprod. 2024 Oct 4;39(12):2830–47. doi: 10.1093/humrep/deae232 (PMC11629989; doi:10.1093/humrep/deae232)

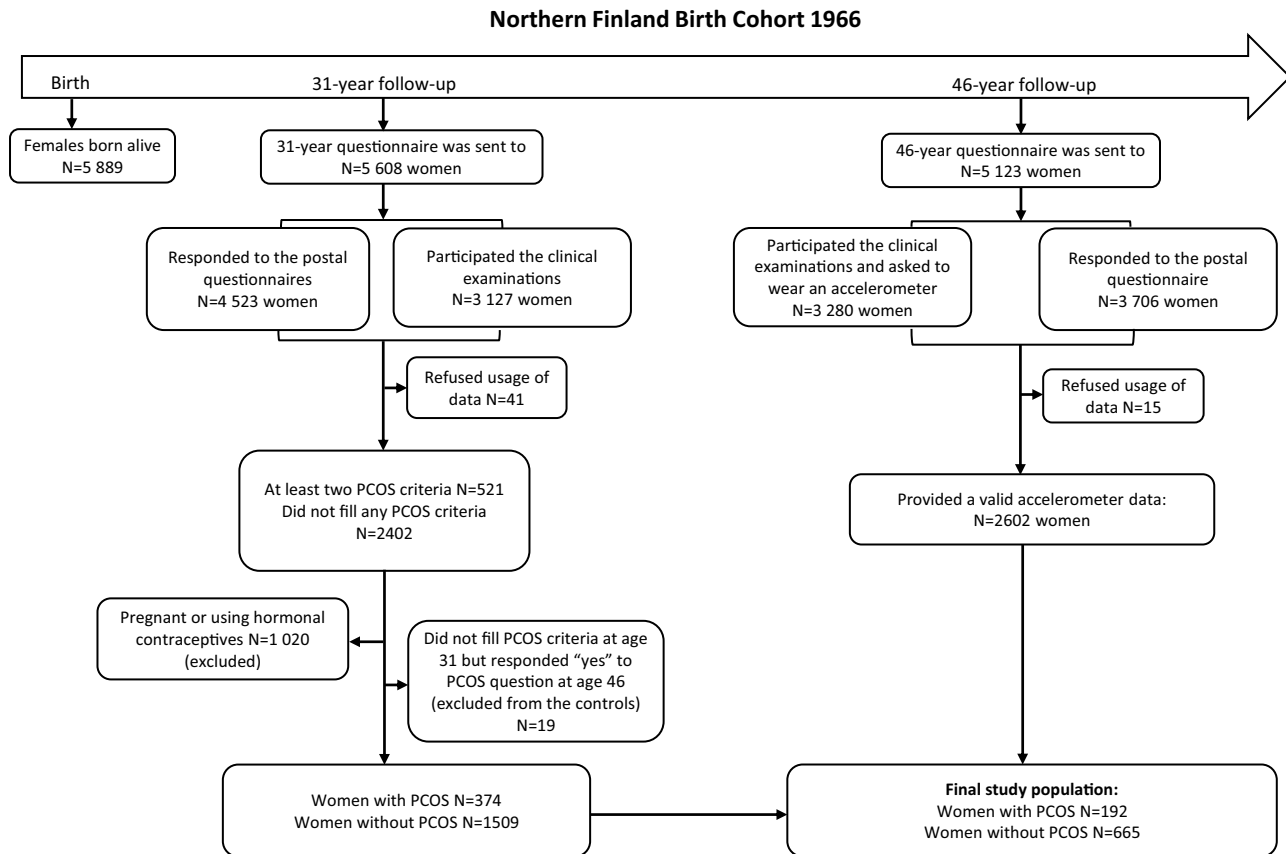

**Supplementary Figure S1. The flowchart of the study.** PCOS: polycystic ovary syndrome.

Supplement: deae232_Supplementary_Figure_S1 [file deae232_supplementary_figure_s1.pdf]
