# Supplementary material for: 24-hour movement behaviours and cardiometabolic markers in women with polycystic ovary syndrome (PCOS): a compositional data analysis
Source: Hum Reprod. 2024 Oct 4;39(12):2830–47. doi: 10.1093/humrep/deae232 (PMC11629989; doi:10.1093/humrep/deae232)
Supplement: deae232_Supplementary_Figure_S2 [file deae232_supplementary_figure_s2.pdf]

OXFORD
human reproduction
Human Reproduction, 2024, 1–1
https://doi.org/10.1093/humrep/deae232
Supplementary Data

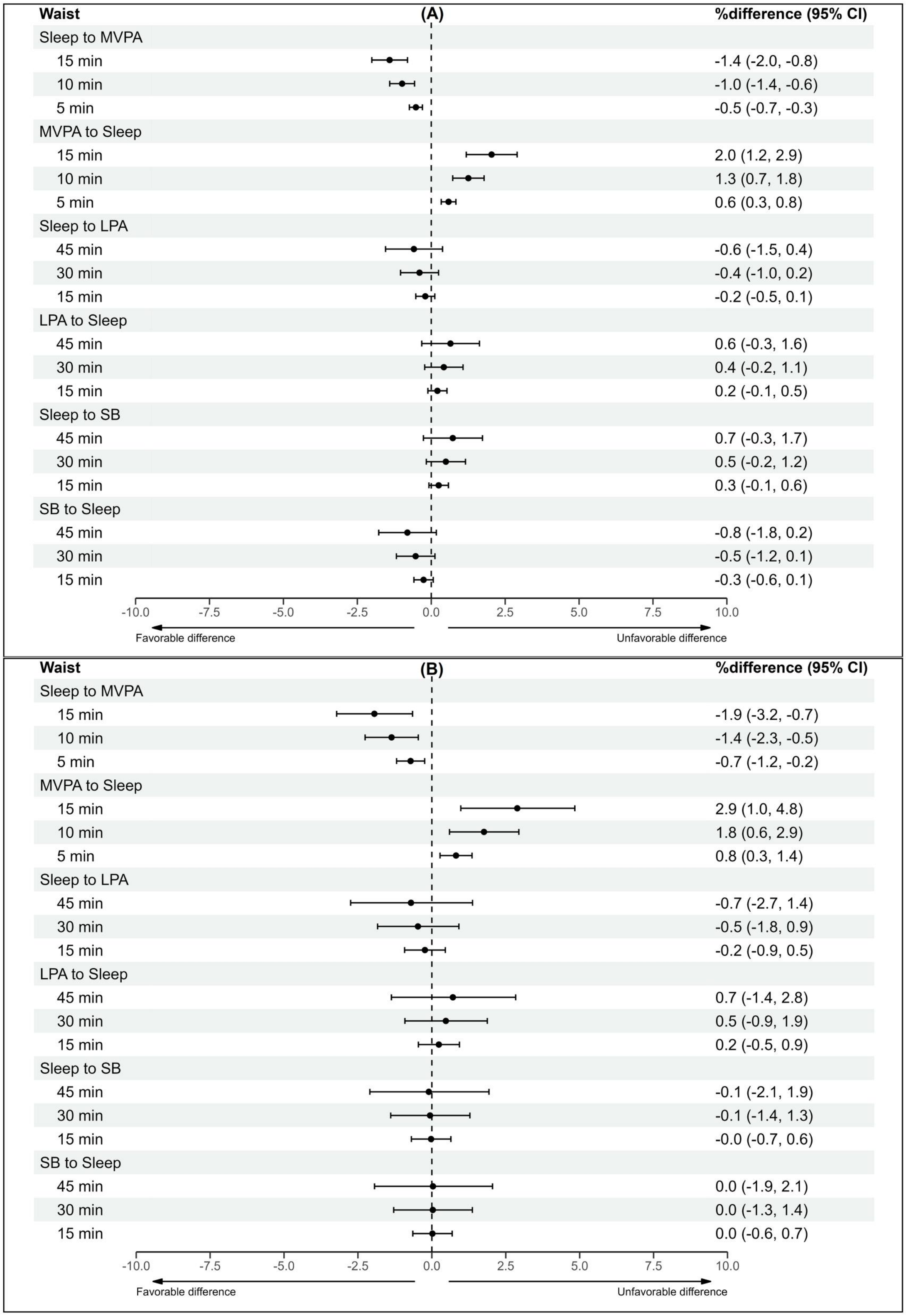

Supplementary Figure S2. The difference in waist circumference with varying 24-h composition. (**A**) Controls and (**B**) women with PCOS. Estimates are expressed as percentage differences (95% CI) when time is reallocated from or to sleep in a pairwise manner within the 24-h movement composition. Adjusted for education, marital status, alcohol use, smoking, psychological distress, and medication use (blood glucose lowering, lipid modifying, or antihypertensive). MVPA, moderate-to-vigorous physical activity; LPA, light physical activity; SB, sedentary behaviour; PCOS, polycystic ovary syndrome.
