# Supplementary material for: 24-hour movement behaviours and cardiometabolic markers in women with polycystic ovary syndrome (PCOS): a compositional data analysis
Source: Hum Reprod. 2024 Oct 4;39(12):2830–47. doi: 10.1093/humrep/deae232 (PMC11629989; doi:10.1093/humrep/deae232)
Supplement: deae232_Supplementary_Table_S1 [file deae232_supplementary_table_s1.pdf]

OXFORD
human reproduction

Human Reproduction, 2024, 1–1
https://doi.org/10.1093/humrep/deae232
Supplementary Data

Supplementary Table S1. The contents and scoring of the Healthy Diet index domains.

Domain	Content
Meal pattern<br>0–10 p	• Eating main meals daily,[a] 0–6 p<br>• Amount of meals and snacks/day,[a] 0–1 p<br>• Variety of main ingredients in meals,[b] 0–3 p
Grains<br>0–20 p	• Rye and multigrain bread,[a] 0–10 p<br>• Whole grain porridge,[b] 0–4 p<br>• Unsweetened muesli,[b] 0–2 p<br>• Dark pasta/rice,[b] 0–1 p<br>• Sweet coffee breads[b] and white bread,[a] 0–3 p
Vegetables and fruits<br>0–16 p → scaled 0–20 p	• Fresh and boiled vegetables,[b] 0–8 p<br>• Berries and fruits,[b] 0–8 p Limitation: no portion sizes
Fats<br>0–11 p → scaled 0–15 p	• Oil-based salad dressing,[b] 0–3 p<br>• Cooking fat,[a] 0–2 p<br>• Bread spread,[a] 0–6 p<br>Limitation: cooking cream, nuts, seeds and almonds are lacking
Fish and meat<br>0–9 p → scaled 0–10 p	• Fish,[b] 0–2 p<br>• Sausages, game, vegetarian food,[b] 0–3 p<br>• Cold cuts,[b] 0–4 p<br>Limitation: beef, pork, and lamb are lacking
Dairy<br>0–10 p	• Low-fat milk and fermented milk,[a] 0–3 p<br>• Medium-fat milk and fermented milk,[a] 0–1 p<br>• Fatty milk,[a] 0–1 p<br>• Cheese slices (by fat content) on bread,[a] 0–5 p
Snacks and treats<br>0–15 p	• Fast food and salty snacks,[b] 0–3 p<br>• Sweet coffee breads, ice cream, and chocolate,[b] 0–3 p<br>• Candy,[b] 0–3 p<br>• Sugar-sweetened drinks[b] and alcohol,[a] 0–6 p<br>Limitation: the use of sugar, honey, juices, and fruit juices are lacking

[a] Based on a detailed question (e.g. information on the quantity and product used).
[b] Based on Food Frequency Questionnaire.
