# Supplementary material for: 24-hour movement behaviours and cardiometabolic markers in women with polycystic ovary syndrome (PCOS): a compositional data analysis
Source: Hum Reprod. 2024 Oct 4;39(12):2830–47. doi: 10.1093/humrep/deae232 (PMC11629989; doi:10.1093/humrep/deae232)
Supplement: deae232_Supplementary_Table_S2 [file deae232_supplementary_table_s2.pdf]

OXFORD
human reproduction
Human Reproduction, 2024, 1–1
https://doi.org/10.1093/humrep/deae232
Supplementary Data

Supplementary Table S2. Univariate associations between the movement behaviours and cardiometabolic markers in controls and women with PCOS.

Cardiometabolic markers	Controls β	Controls 95% CI	Controls P-value	PCOS β	PCOS 95% CI	PCOS P-value	P-value int.[a]
**MVPA**							
Waist circumference	−0.25	−0.32 to −0.17	**<0.001**	−0.27	−0.41 to −0.12	**<0.001**	0.654
Triglycerides	−0.16	−0.23 to −0.08	**<0.001**	−0.24	−0.38 to −0.09	**0.002**	0.429
fp-glucose	−0.16	−0.24 to −0.08	**<0.001**	−0.11	−0.26 to 0.04	0.166	0.775
fs-insulin	−0.19	−0.27 to −0.12	**<0.001**	−0.31	−0.46 to −0.17	**<0.001**	0.141
HOMA-IR	−0.18	−0.25 to −0.10	**<0.001**	−0.24	−0.39 to −0.09	**0.002**	0.385
2-h glucose*	−0.14	−0.22 to −0.05	**0.001**	−0.05	−0.21 to 0.11	0.535	0.321
2-h insulin	−0.24	−0.32 to −0.16	**<0.001**	−0.13	−0.29 to 0.04	0.124	0.166
hs-CRP	−0.20	−0.28 to −0.12	**<0.001**	−0.17	−0.32 to −0.02	**0.031**	0.684
MAP	−0.14	−0.22 to −0.07	**<0.001**	−0.10	−0.25 to 0.05	0.175	0.658
**LPA**							
Waist circumference	−0.12	−0.21 to −0.03	**0.008**	−0.03	−0.20 to 0.15	0.771	0.389
Triglycerides	−0.17	−0.26 to −0.08	**<0.001**	−0.11	−0.28 to 0.07	0.226	0.552
fp-glucose	−0.09	−0.18 to 0.00	0.059	−0.06	−0.24 to 0.12	0.520	0.956
fs-insulin	−0.16	−0.25 to −0.06	**<0.001**	−0.06	−0.23 to 0.11	0.506	0.397
HOMA-IR	−0.16	−0.25 to −0.07	**<0.001**	−0.06	−0.24 to 0.12	0.515	0.401
2-h glucose*	0.008	−0.14 to 0.12	0.901	0.03	−0.17 to 0.22	0.781	0.808
2-h insulin	−0.08	−0.18 to 0.02	0.101	0.02	−0.18 to 0.21	0.855	0.387
hs-CRP	−0.06	−0.15 to 0.04	0.235	0.09	−0.09 to 0.27	0.333	0.156
MAP	−0.03	−0.13 to 0.06	0.493	0.13	−0.04 to 0.31	0.138	0.092
**SB**							
Waist circumference	0.21	0.09 to 0.33	**<0.001**	0.06	−0.17 to 0.29	0.617	0.229
Triglycerides	0.15	0.03 to 0.27	**0.014**	0.03	−0.20 to 0.26	0.784	0.327
fp-glucose	0.08	−0.04 to 0.21	0.182	0.12	−0.13 to 0.36	0.346	0.688
fs-insulin	0.18	0.06 to 0.30	**0.004**	−0.02	−0.25 to 0.21	0.888	0.128
HOMA-IR	0.15	0.03 to 0.27	**0.015**	0.03	−0.21 to 0.27	0.812	0.343
2-h glucose*	0.10	−0.07 to 0.26	0.259	−0.03	−0.29 to 0.23	0.809	0.321
2-h insulin	0.15	0.02 to 0.27	**0.021**	−0.06	−0.32 to 0.19	0.637	0.132
hs-CRP	0.16	0.04 to 0.28	**0.010**	0.06	−0.18 to 0.29	0.628	0.428
MAP	0.13	0.01 to 0.26	**0.032**	0.13	−0.11 to 0.37	0.283	0.952
**Sleep**							
Waist circumference	0.05	−0.06 to 0.17	0.387	0.09	−0.13 to 0.32	0.413	0.704
Triglycerides	0.09	−0.03 to 0.21	0.129	0.16	−0.06 to 0.38	0.159	0.628
fp-glucose	0.08	−0.04 to 0.20	0.216	0.02	−0.22 to 0.26	0.878	0.714
fs-insulin	0.08	−0.04 to 0.19	0.205	0.18	−0.04 to 0.41	0.111	0.392
HOMA-IR	0.09	−0.03 to 0.21	0.148	0.13	−0.10 to 0.36	0.277	0.738
2-h glucose*	−0.001	−0.18 to 0.18	0.999	0.02	−0.24 to 0.28	0.888	0.812
2-h insulin	0.06	−0.06 to 0.18	0.318	0.07	−0.18 to 0.33	0.560	0.931
hs-CRP	0.02	−0.10 to 0.14	0.775	−0.04	−0.28 to 0.19	0.720	0.644
MAP	−0.007	−0.13 to 0.11	0.911	−0.15	−0.38 to 0.08	0.210	0.271

The associations between each movement behaviour and cardiometabolic marker are expressed in relation to the rest of the 24-h movement composition. Standardized beta coefficients (β) are presented with 95% CI. Statistically significant associations ($P < 0.05$) are bolded.

[a] P-value interaction indicates whether the association between movement behaviour and cardiometabolic marker is statistically different between controls and women with PCOS.

* In controls, showed a U-shaped relationship with mean sleep duration (<8.5 h and ≥8.5 h).

PCOS, polycystic ovary syndrome; fp-glucose, fasting plasma glucose; fs-insulin, fasting serum insulin; HOMA-IR, The Homeostatic Model Assessment–insulin resistance; hs-CRP, The high-sensitivity C-reactive protein; MAP, mean arterial pressure; MVPA, moderate-to-vigorous physical activity; LPA, light physical activity; SB, sedentary behaviour.
