# Supplementary material for: 24-hour movement behaviours and cardiometabolic markers in women with polycystic ovary syndrome (PCOS): a compositional data analysis
Source: Hum Reprod. 2024 Oct 4;39(12):2830–47. doi: 10.1093/humrep/deae232 (PMC11629989; doi:10.1093/humrep/deae232)
Supplement: deae232_Supplementary_Table_S3 [file deae232_supplementary_table_s3.pdf]

OXFORD

human reproduction

Human Reproduction, 2024, 1–1
https://doi.org/10.1093/humrep/deae232
Supplementary Data

Supplementary Table S3. Associations between the movement behaviours and 2-h glucose in controls stratified by the mean sleep duration.

	Sleep duration < 8.5						Sleep duration ≥ 8.5					
**Movement and non-movement behaviours**	**n**	**Model R^2**	**Model *p***	**β**	**95% CI**	***P*-value**	**n**	**Model R^2**	**Model *P***	**β**	**95% CI**	***P*-value**
**Univariate model**												
MVPA	245	0.02	0.239	−0.001	−0.14 to 0.16	0.922	341	0.05	<0.001	−0.23	−0.34 to -0.12	**<0.001**
LPA	245	0.02	0.239	0.23	−0.18 to 0.64	0.271	341	0.05	<0.001	−0.24	−0.52 to 0.05	0.106
SB	245	0.02	0.239	0.46	−0.10 to 1.01	0.105	341	0.05	<0.001	−0.25	−0.64 to 0.15	0.223
Sleep	245	0.02	0.239	−0.33	−0.81 to 0.15	0.176	341	0.05	<0.001	0.34	−0.03 to 0.72	0.073
**Adjusted model**												
MVPA	232	0.12	0.004	0.03	−0.13 to 0.18	0.731	312	0.10	<0.001	−0.21	−0.33 to −0.09	**<0.001**
LPA	232	0.12	0.004	0.16	−0.26 to 0.58	0.448	312	0.10	<0.001	−0.20	−0.49 to 0.10	0.185
SB	232	0.12	0.004	0.49	−0.07 to 1.04	0.085	312	0.10	<0.001	−0.13	−0.55 to 0.29	0.541
Sleep	232	0.12	0.004	−0.31	−0.79 to 0.17	0.202	312	0.10	<0.001	0.25	−0.14 to 0.65	0.203

The associations between each movement and non-movement behaviour and 2-h glucose are expressed in relation to the rest of the 24-h movement composition. Standardized beta coefficients (β) are presented with 95% CI. Statistically significant associations ($P < 0.05$) are bolded. The adjusted model includes adjustments for education, marital status, alcohol use, smoking, psychological distress, and medication use (blood glucose lowering, lipid modifying, or antihypertensive). MVPA, moderate-to-vigorous physical activity; LPA, light physical activity; SB, sedentary behaviour.
