# Supplementary material for: 24-hour movement behaviours and cardiometabolic markers in women with polycystic ovary syndrome (PCOS): a compositional data analysis
Source: Hum Reprod. 2024 Oct 4;39(12):2830–47. doi: 10.1093/humrep/deae232 (PMC11629989; doi:10.1093/humrep/deae232)
Supplement: deae232_Supplementary_Table_S4 [file deae232_supplementary_table_s4.pdf]

OXFORD
human reproduction
Human Reproduction, 2024, 1–1
https://doi.org/10.1093/humrep/deae232
Supplementary Data

Supplementary Table S4. Compositional means of behaviours within 24-h cycle in PCOS-phenotypes.

Movement behaviours	A + B-phenotype (n = 62)	C-phenotype (n = 62)	D-phenotype (n = 68)
MVPA	43.7 (2.6%)	45.7 (2.7%)	45.7 (2.9%)
LPA	391.2 (27.0%)	395.9 (27.4%)	399.9 (27.6%)
SB	490.7 (34.1%)	478.4 (33.2%)	463.5 (32.1%)
Sleep	514.4 (36.3%)	519.9 (36.7%)	530.9 (37.4%)

Compositional means are expressed as minutes and as percentages of a 1440 min (24-h) day. A + B-phenotype (PCOS-classic): A-phenotype (HA + OA + AMH) or B-phenotype (HA + OA). PCOS-C: C-phenotype (HA + AMH). PCOS-D: D-phenotype (OA + AMH).
PCOS, polycystic ovary syndrome; MVPA, moderate-to-vigorous physical activity; LPA, light physical activity; SB, sedentary behaviour; HA, hyperandrogenism; OA, oligo/amenorrhoea; AMH, anti-Müllerian hormone.
