# Supplementary material for: 24-hour movement behaviours and cardiometabolic markers in women with polycystic ovary syndrome (PCOS): a compositional data analysis
Source: Hum Reprod. 2024 Oct 4;39(12):2830–47. doi: 10.1093/humrep/deae232 (PMC11629989; doi:10.1093/humrep/deae232)
Supplement: deae232_Supplementary_Table_S5 [file deae232_supplementary_table_s5.pdf]

OXFORD
human reproduction
Human Reproduction, 2024, 1–1
https://doi.org/10.1093/humrep/deae232
Supplementary Data

Supplementary Table S5. Univariate associations between overall 24-h movement composition and cardiometabolic markers in PCOS-phenotypes.

	A + B-phenotype			C-phenotype			A + B and C-phenotype (HA-phenotype)			D-phenotype (non-HA-phenotype)		
**Cardiometabolic markers**	**n**	**Model R^2**	**Model P**	**n**	**Model R^2**	**Model P**	**n**	**Model R^2**	**Model P**	**n**	**Model R^2**	**Model P**
Waist circumference	62	0.11	0.090	62	0.10	0.103	124	0.09	**0.013**	67	0.17	**0.008**
Triglycerides	62	0.02	0.769	62	0.18	**0.010**	124	0.07	**0.033**	68	0.17	**0.007**
fp-glucose	62	0.02	0.734	59	0.13	0.051	121	0.05	0.134	65	0.08	0.168
fs-insulin	60	0.06	0.323	61	0.10	0.101	121	0.08	**0.023**	66	0.34	**<0.001**
HOMA-IR	59	0.02	0.720	57	0.09	0.164	116	0.05	0.125	64	0.28	**<0.001**
2-h glucose	51	0.02	0.764	55	0.07	0.306	106	0.01	0.789	57	0.03	0.633
2-h insulin	52	0.004	0.978	56	0.11	0.120	108	0.03	0.354	57	0.19	**<0.001**
hs-CRP	61	0.06	0.335	61	0.09	0.150	122	0.05	0.090	64	0.05	0.418
MAP	62	0.01	0.832	61	0.12	0.058	123	0.04	0.191	68	0.01	0.903

The table includes R^2 values and statistical significances for each compositional model created for each cardiometabolic marker (model P-value). Statistically significant associations ($P < 0.05$) are bolded. A + B-phenotype (PCOS-classic): A-phenotype (HA + OA + AMH) or B-phenotype (HA + OA). PCOS-C: C-phenotype (HA + AMH). PCOS-D: D-phenotype (OA + AMH).
PCOS, polycystic ovary syndrome; fp-glucose, fasting plasma glucose; fs-insulin, fasting serum insulin; HOMA-IR, The Homeostatic Model Assessment–insulin resistance; hs-CRP, The high-sensitivity C-reactive protein; MAP, mean arterial pressure; HA, hyperandrogenism; OA, oligo/amenorrhoea; AMH, anti-Müllerian hormone.

© The Author(s) 2024. Published by Oxford University Press on behalf of European Society of Human Reproduction and Embryology.
This is an Open Access article distributed under the terms of the Creative Commons Attribution License (https://creativecommons.org/licenses/by/4.0/), which permits unrestricted reuse, distribution, and reproduction in any medium, provided the original work is properly cited.
Human Reproduction, 2024, 00, 1–28
https://doi.org/10.1093/humrep/deae232
Original Article
